# Hierarchically Porous 3D Freestanding Holey-MXene Framework via Mild Oxidation of Self-Assembled MXene Hydrogel for Ultrafast Pseudocapacitive Energy Storage

**DOI:** 10.1021/acsnano.3c11551

**Published:** 2024-01-17

**Authors:** Anirban Sikdar, Frédéric Héraly, Hao Zhang, Stephen Hall, Kanglei Pang, Miao Zhang, Jiayin Yuan

**Affiliations:** † Department of Materials and Environmental Chemistry (MMK), Stockholm University, 10691 Stockholm, Sweden; ‡ Division of Solid Mechanics, Lund University, 22100 Lund, Sweden

**Keywords:** self-assembly, 2D materials, holey-MXene, freestanding hydrogel, pseudocapacitor

## Abstract

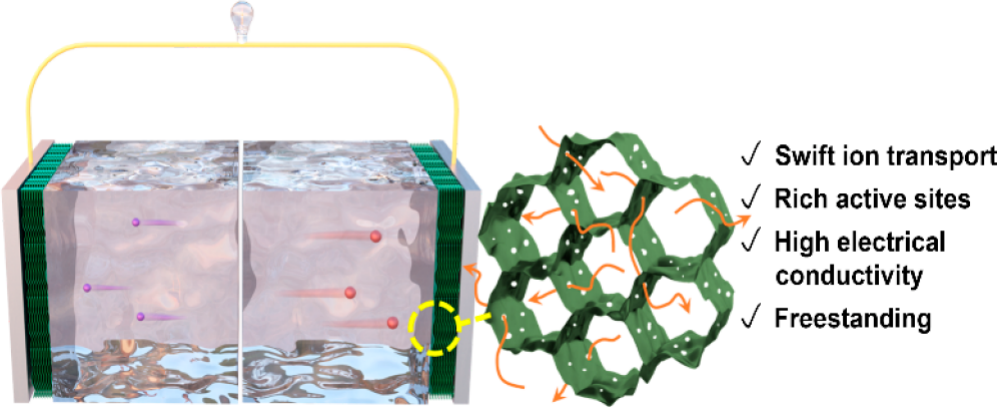

The true promise of MXene as a practical supercapacitor electrode hinges on the simultaneous advancement of its three-dimensional (3D) assembly and the engineering of its nanoscopic architecture, two critical factors for facilitating mass transport and enhancing an electrode’s charge-storage performance. Herein, we present a straightforward strategy to engineer robust 3D freestanding MXene (Ti_3_C_2_T_*x*_) hydrogels with hierarchically porous structures. The tetraamminezinc(II) complex cation ([Zn(NH_3_)_4_]^2+^) is selected to electrostatically assemble colloidal MXene nanosheets into a 3D interconnected hydrogel framework, followed by a mild oxidative acid-etching process to create nanoholes on the MXene surface. These hierarchically porous, conductive holey-MXene frameworks facilitate 3D transport of both electrons and electrolyte ions to deliver an excellent specific capacitance of 359.2 F g^–1^ at 10 mV s^–1^ and superb capacitance retention of 79% at 5000 mV s^–1^, representing a 42.2% and 15.3% improvement over pristine MXene hydrogel, respectively. Even at a commercial-standard mass loading of 10.1 mg cm^–2^, it maintains an impressive capacitance retention of 52% at 1000 mV s^–1^. This rational design of an electrode by engineering nanoholes on MXene nanosheets within a 3D porous framework dictates a significant step forward toward the practical use of MXene and other 2D materials in electrochemical energy storage systems.

Supercapacitors represent a promising technology for electrochemical energy storage due to their ultrahigh power density and long cycle life. Over the past decade, there has been significant progress in the development of high-performing electrodes for supercapacitor applications, which include various two-dimensional (2D) nanomaterials,^1,2^ conducting polymers,^3,4^ transition metal oxides/hydroxides,^5,6^ their hybrids, and beyond.^7−12^ Recently, 2D transition metal carbides, nitrides, or carbonitrides, also known as MXenes, have shown tremendous potential for energy storage applications due to their excellent electrical conductivity, largely accessible surface area, surface redox activity, and the capability of colloidal assembly.^13,14^ Nevertheless, a rational design of the MXene architecture from a nanoscopic level to macroscopic assemblies is crucial in harnessing their favorable physicochemical properties for practical use in energy storage systems.

Lately, the assembly of MXene nanosheets into 3D interconnected porous hydrogel structures has emerged as a promising approach to develop freestanding supercapacitor electrodes.^15−17^ These hydrogels can potentially combine the intrinsic charge-storage capability of MXenes with a 3D porous structure that facilitates swift transport of both electrons through the solid framework and electrolyte ions via the liquid phase. Despite previous efforts to employ 3D MXene hydrogels as supercapacitor electrodes, their widespread implementation, especially for high-rate performance supercapacitors, faces critical challenges.

First, a primary challenge lies in the development of a truly freestanding and MXene-exclusive hydrogel. The difficulty arises from the lack of methods to uniformly gelate the MXene dispersion due to their weak intersheet interactions, inherent surface rigidity, and tendency to restack during assembly. Consequently, most reported freestanding MXene hydrogels to date have included nonconductive polymers or chemically modified graphene as binders or linkers.^18−23^ These additives, while aiding structural integrity, may compromise the charge-storage performance of the composite MXene hydrogel. Recently, a few reports have demonstrated a straightforward ion-induced gelation strategy for MXene.^24−26^ However, the vulnerable mechanical performance of these MXene hydrogels could hardly enable complete free-standing characteristics, thus often requiring an additional energy-intensive and time-consuming freeze-drying step to meet the necessary mechanical strength.

Second, at present, there is a lack of significant advances in the optimal design of the pristine MXene-hydrogel-based electrode architecture. As a result, the rate performance of most of the previously reported MXene-hydrogel-based electrodes has been far from satisfactory, often deteriorating rapidly, especially at scan rates above 1000 mV s^–1^.^23,27,28^ This issue becomes even more severe as the electrode’s mass loading approaches the commercial standard (∼10 mg cm^–2^).^29−31^ Their poor rate performance is primarily associated with the increased ion diffusion resistance, a consequence of the long and tortuous diffusion channels in inadequately designed electrodes. Therefore, MXene hydrogels with favorable mass transport pathways need to be designed judiciously to maintain high energy storage performance even at a commercial mass loading.

To tackle the aforementioned challenges, herewith we established a colloidal approach to assemble 3D freestanding holey-MXene hydrogels that are applied as high-performance supercapacitor electrodes. The hydrogel networks were prepared by cation-induced electrostatic assembly of colloidal MXene nanosheets, followed by a mild oxidative acid-etching process to generate nanoholes on the MXene surface as extra pores. Such hydrogels display superb electrochemical performance, achieving a specific capacitance of 359.2 F g^–1^ at 10 mV s^–1^ and maintaining up to 79% of this capacitance at an ultrafast scan rate of 5000 mV s^–1^, surpassing the pristine MXene hydrogel by 42.2% and 15.3%, respectively. Besides, at an electrode mass loading of 10.1 mg cm^–2^, which corresponds to the commercial standard, the hydrogel retains over 52% of its initial capacitance at a scan rate of 1000 mV s^–1^. The excellent capacitive performance is further reflected by its capability of delivering an ultrahigh energy density of 11.4 Wh kg^–1^ at a power density of 410 W kg^–1^ in a symmetric device configuration. Such a promising electrochemical performance lies in the engineered nanoholes of tunable sizes on the MXene surface in the hydrogel and the presence of a stable and freestanding conductive MXene network without any insulating binders or additives.

## Results and Discussion

### Fabrication and Characterizations of Hydrogels

**Figure 1:**
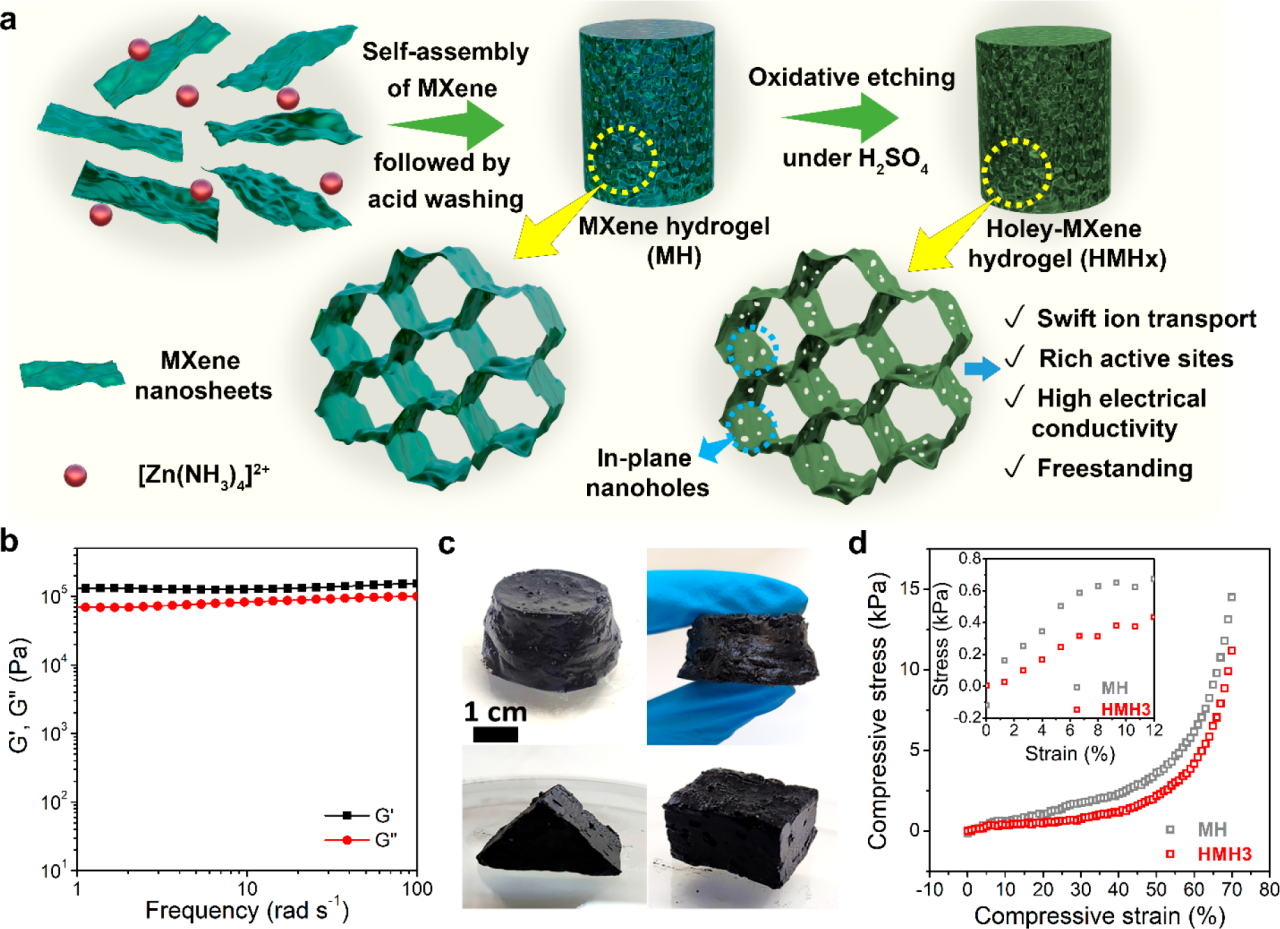
**Schematic of the fabrication process and the properties of MXene hydrogels (MH) and holey-MXene hydrogels (HMHx).** (a) Schematic of the fabrication procedure for MH and HMHx. The assembly of a MXene nanosheet into a freestanding hydrogel is induced by the electrostatic interaction between the divalent [Zn(NH_3_)_4_]^2+^ cations and MXene; after a mild oxidative etching process by 3 M H_2_SO_4_ acid, the holey-MXene hydrogel is fully developed. (b) Rheological properties of MH displaying typical hydrogel characteristics. (c) Digital images of HMH3 in different shapes. (d) Compressive strain–stress curves of aerogels made from MH and HMH3. The inset in (d) shows the elastic regime of the stress–strain curve.

Figure 1a schematically illustrates the fabrication procedure of MXene hydrogels (MH) and holey-MXene hydrogels (HMHx, where “x” denotes the duration of the oxidative acid-etching process in hours) and the comparison of their morphological features. Freestanding MXene hydrogels were prepared by the aqueous assembly of colloidal Ti_3_C_2_T_*x*_ MXene nanosheets, initiated by the divalent tetraamminezinc(II) complex cation ([Zn(NH_3_)_4_]^2+^). Detailed preparation procedures can be found in the Experimental Section. Briefly, an aqueous dispersion of Ti_3_C_2_T_*x*_ MXene was first obtained by etching and delaminating a Ti_3_AlC_2_ MAX powder in a LiF/HCl mixture solution, following the well-established minimally intensive layer delamination (MILD) protocol.^32^ The MXene dispersion was gelated at a concentration of 10 mg mL^–1^ by adding an aqueous [Zn(NH_3_)_4_](OH)_2_ solution. Driven by the electrostatic attraction between divalent [Zn(NH_3_)_4_]^2+^ cations and negatively charged MXene nanosheets, the colloidal MXene sheets started to order themselves immediately after the addition of the [Zn(NH_3_)_4_](OH)_2_ solution, thus forming a 3D hydrogel framework. Subsequently, the hydrogel was immersed in a 3 M aqueous H_2_SO_4_ solution at ambient temperature to extract all of the zinc species (discussed in a later section), creating pristine MHs. Figure S1a,b and Figure S2a show the digital images of the MXene dispersion and the as-prepared MH. Rheological analysis of MH (Figure 1b) reveals a typical elastic response with a storage modulus (*G*′) higher than the loss modulus (*G*″). Furthermore, both *G*′ and *G*″ remain frequency-independent in the plateau region over the measured frequency range (100–1 rad s^–1^), suggesting the hydrogel-like characteristics of MH. The as-prepared MH serves as the foundational material for the subsequent development of HMHx for high-performance supercapacitor electrodes.

To develop HMHx, we employed a straightforward, mild oxidative acid-etching process. The as-prepared MH was subjected to 3 M H_2_SO_4_ at 60 °C for a defined period, as schematically shown in Figure 1a.^33,34^ During this acid-etching process, in-plane nanoholes were generated on the surface of MXene nanosheets preorganized in a hydrogel network (schematic in Figure 1a). A piece of as-prepared HMHx in acid is shown in Figure S2b. We studied three samples with different etching periods, namely, HMH1, HMH3, and HMH6, to comprehensively assess the impact of etching time on the size and distribution of the in-plane nanoholes on the holey-MXene nanosheets. For most of the remaining characterizations, we used HMH3 as a model since it exhibited the best electrochemical performance among the three HMHx (as discussed later). Figure 1c illustrates the inherent free-standing nature of HMH3 with arbitrary shapes. Its mechanical robustness is further evaluated through compressive stress–strain tests of the aerogels derived from the respective hydrogels (Figure 1d and inset). During the compression, the stress–strain relationship of the aerogels displays a linear elastic regime at the low strain regime (0–8%) (inset of Figure 1d). In the second stage (up to 40%), a stress plateau regime is evident, which is possibly due to the gradual collapse of the macroporous structures in the aerogel. Finally (>40%), a densification stage ensues, marked by an abrupt increase in stress due to the densification of the aerogels caused by the continuous compressive deformation of the porous structure.^35^ Notably, a slight decrease in compressive stress for HMH3 compared to that for MH indicates a minor structural modification when transferring MH to HMH3. Understandably, the acid etching of MH and the corresponding introduction of in-plane nanoholes made HMH3 slightly more brittle, leading to this slight mechanical downgrade.

**Figure 2:**
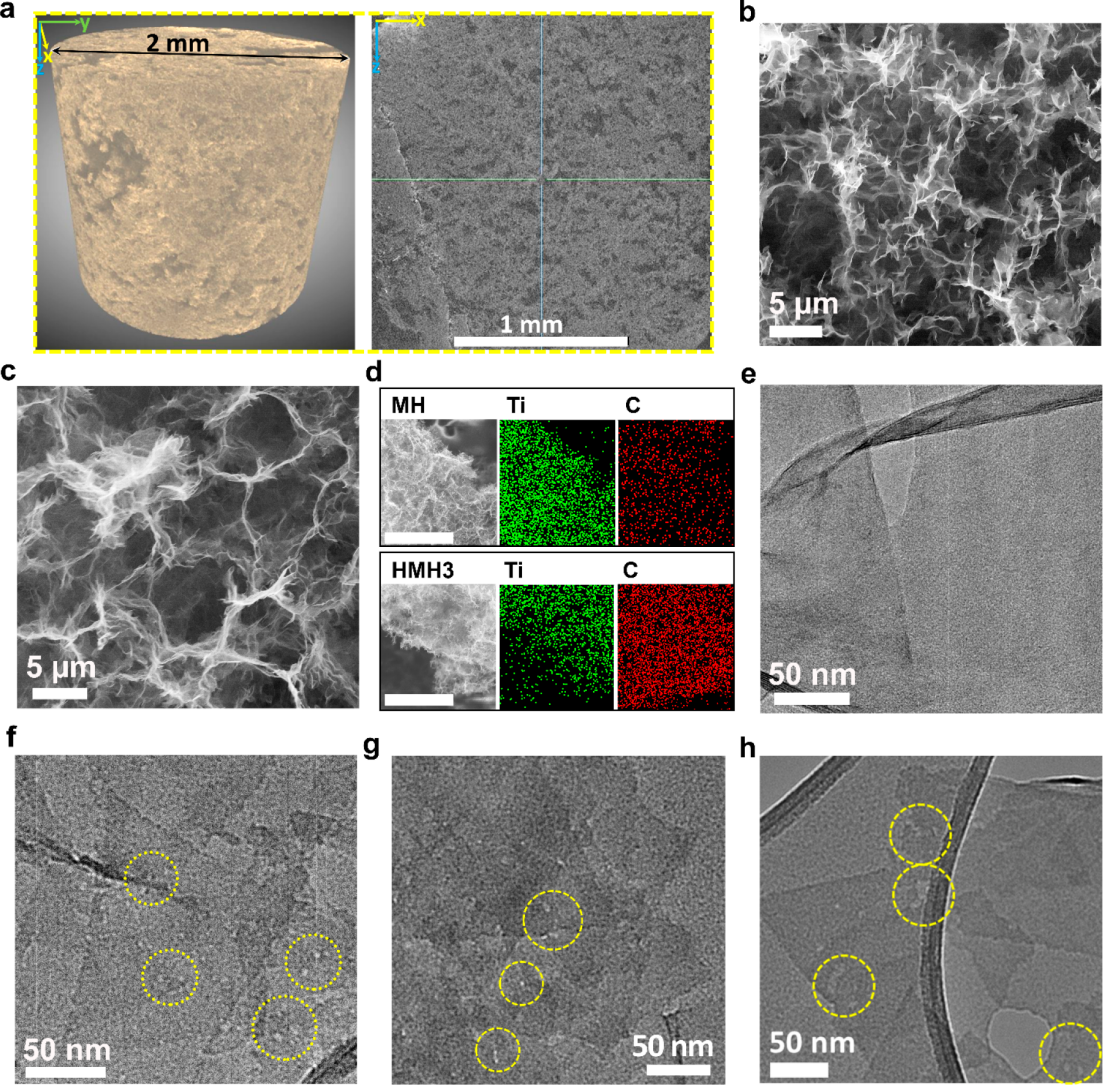
**Characterizations of MH and HMHx.** (a) 3D rendering of the micro-CT profile of freeze-dried HMH3 (left) and a vertical cross-section slice through the center of the micro-CT volume (right). SEM images of freeze-dried samples of (b) MH and (c) HMH3. (d) Elemental mapping of MH and HMH3 via EDX (scale bar: 50 μm). (e–h) TEM images of MH, HMH3, HMH1, and HMH6, respectively. The circular regions in the TEM images (f–h) highlight the locations of in-plane nanoholes.

To gain insights into the morphological features of the as-prepared hydrogels, we employed microcomputed tomography (micro-CT), scanning electron microscopy (SEM), and transmission electron microscopy (TEM) imaging techniques on MH and HMHx. The 3D rendering of the micro-CT profile of freeze-dried HMH3 provides a direct view of the hydrogel scaffolds with interconnected features. Large voids, some up to ∼400 μm in size, are spotted in its direct visualization (Figure 2a), which is induced by the rapid gelation process. A vertical cross-section slice through the center of the micro-CT volume (Figure 2a, right) also exhibits similar porous morphology, confirming the presence of large pores throughout the hydrogel structure.

SEM images of the freeze-dried samples offer detailed insights into the microstructure of the hydrogels. SEM images of freeze-dried MH and HMH3 (Figure 2b and c) reveal aggregation-free porous and interconnected network structures with pore walls composed of MXene nanosheets. The pore sizes in both MH and HMH3 span from submicrometer to up to 10 μm, suggesting that the mild etching treatment of MH in H_2_SO_4_ at 60 °C does not noticeably alter the microstructure of the hydrogel in HMH3. Energy dispersive X-ray (EDX) spectroscopy mapping images of MH and HMH3 (Figure 2d) demonstrate the presence of titanium (Ti) and carbon (C) elements all over the samples, confirming the homogeneous distribution of MXene (Ti_3_C_2_T_*x*_) nanosheets in the hydrogels. Moreover, the water contents in MH and HMH3 were found to be about 99% and 97% from thermogravimetric (TG) analysis (Figure S3), respectively, suggesting that the hydrogel property remains intact in HMH3.

To further reveal the microstructure of the hydrogels and the formation of nanoholes on the surface of holey-MXene nanosheets, we conducted TEM analysis. Low- and high-magnification TEM images of MH (Figure S4, Figure 2e) display a wrinkled structure of MXene nanosheets with a clean surface texture. The clean surface, without the presence of TiO_2_ nanoparticles, evidences no noticeable oxidation of MXene during the gelation process. Importantly, the TEM image of HMH3 (Figure 2f) confirms the formation of nanoholes distributed across the surface of the MXene nanosheets with an average pore size of 3.2 ± 0.15 nm, suggesting the presence of holey-MXene nanosheets in HMH3. We also examined the microstructures of HMH1 and HMH6 using SEM and TEM images to elucidate the effect of etching time on the porous structure of the hydrogels. It was found that the overall porous network of the hydrogels remained essentially unchanged as the etching time increased from 1 h in HMH1 to 3 h in HMH3 and further to 6 h in HMH6, as indicated by the SEM images in Figure 2b,c and Figure S5a,b. However, the TEM images of these hydrogels (Figure 2f–h) revealed that the average size of the in-plane nanoholes on holey-MXene nanosheets increased from 2.2 ± 0.2 nm in HMH1 to 9.0 ± 1.3 nm in HMH6, indicating that the time of acid-etching plays a crucial role in controlling the size of the nanoholes on the holey-MXene nanosheets.

To further corroborate the porous microstructure of the hydrogels, we studied Brunauer–Emmett–Teller (BET) specific surface areas (SSAs) and pore size distributions. Nitrogen adsorption/desorption isotherms recorded at 77 K (Figure S6a) present narrow hysteresis loops for both MH and HMH3, which are identified as type II isotherms.^36^ The analysis of the pore size distribution (Figure S6b) indicates that the freeze-dried hydrogels are predominantly composed of mesopores ranging in size from 2 to 10 nm. The SSAs of MH and HMH3, determined from the BET equation, were calculated to be 20 and 29 m^2^ g^–1^, respectively. These values of SSAs are comparable to those of other reported MXene-based porous materials.^37,38^

**Figure 3:**
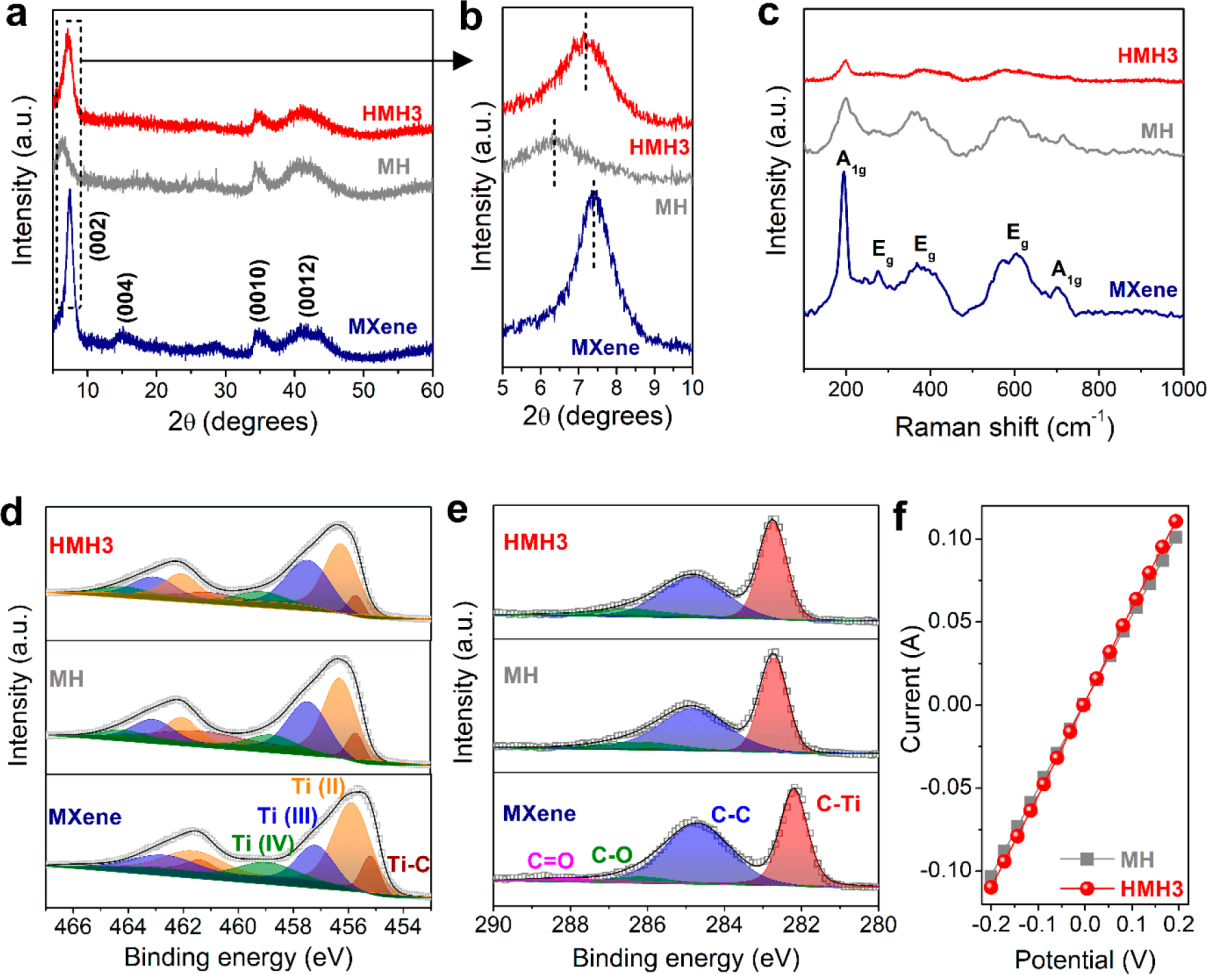
**Structural characterizations of MH and HMHx.** (a) XRD patterns of pure MXene, MH, and HMH3. (b) The enlarged view of the XRD patterns showing the difference in the peak positions for MXene, MH, and HMH3. (c) Raman spectra and (d) high-resolution Ti 2p and (e) high-resolution C 1s spectra of pure MXene, MH, and HMH3. (f) Comparison of the electrical conductivities of MH and HMH3.

The phase structure of the aerogels derived from hydrogels was studied by an X-ray diffraction technique. Upon delamination of Ti_3_AlC_2_ MAX into MXene, most of the peaks assigned to Ti_3_AlC_2_ MAX disappeared, and a prominent diffraction peak at 7.2° emerged (Figure 3a and Figure S7). This peak corresponds to the (002) crystallographic plane with an interlayer spacing of 1.22 nm, typical for Ti_3_C_2_T_*x*_ MXene.^39^ As depicted in Figure 3a, the XRD patterns of the freeze-dried MH and HMH3 exhibit characteristics similar to those of pure MXene nanosheets. However, a closer look at the (002) diffraction peaks for different MXene samples (Figure 3b) reveals that, after the formation of the hydrogel, the (002) diffraction peak at 7.2° for pure MXene nanosheets downshifts to 6.3° for MH. This change suggests an increase of the interlayer spacing from 1.22 nm for pure MXene to 1.40 nm for MH, possibly due to the interleaving of [Zn(NH_3_)_4_]^2+^ cations and water molecules between MXene layers during the formation of the hydrogel. Interestingly, in HMH3, the (002) diffraction peak upshifts to 7.1°, corresponding to a decreased interlayer spacing of 1.24 nm (Figure 3b). It indicates structural changes in the MXene nanosheets when transferring from MH to HMH3 as a result of the thermal acid-etching process. Moreover, the broadening of the peaks in both MH and HMH3 in comparison to pure MXene (Figure 3b) suggests an increase in the disordered organization of the MXene nanosheets,^38^ which is consistent with the porous network structure of the hydrogels, as confirmed by the micro-CT, SEM, and gas sorption analysis.

To investigate the structural characteristics and chemical bonding of MXene and different MXene hydrogels, Raman spectroscopy analysis was carried out. The Raman spectrum of pure MXene showcases characteristic Raman modes centered at 195.2 and 702 cm^–1^, which are ascribed to the A_1g_ symmetric out-of-plane vibrations of Ti and C atoms, respectively (Figure 3c).^40^ Besides, the Raman bands observed at 276.6, 367.9, and 603.6 cm^–1^ are attributed to the in-plane vibrations (E_g_ group) of Ti, C, and atoms in the surface functional groups. The Raman spectra of MH and HMH3 exhibit similar bands to those of pure MXene. However, broadening of the Raman modes in MH and HMH3 as compared to pure MXene is identified and can be attributed to the interaction between MXene nanosheets and their disordered arrangement in the hydrogel structure.^41^ Moreover, no vibrational mode originating from TiO_2_ (characteristic band at ∼144 cm^–1^) was detected in MH and HMH3, ruling out the detectable oxidation of MXene nanosheets in these hydrogels.^42,43^ These results confirm that the chemical structure of MXene in the hydrogels is preserved well following gelation and the introduction of nanoholes.

X-ray photoelectron spectroscopy (XPS) analysis was applied to examine the surface chemical properties of the MXene hydrogels. The XPS survey spectra of pure MXene, MH, and HMH3 (Figure S8) suggest the existence of Ti, C, oxygen (O), and fluorine (F) elements in all the samples. No characteristic peak associated with Zn was observed in the survey spectra of MH and HMH3, illustrating the quantitative removal of Zn species after acid treatment.

The high-resolution Ti 2p core level spectrum of pure MXene, as depicted in Figure 3d, can be deconvoluted into four doublets (Ti 2p_3/2_–Ti 2p_1/2_), of which the Ti 2p_3/2_ components centered at 455.2, 455.9, 457.2, and 459.0 eV correspond to Ti—C, Ti (II), Ti (III), and Ti (IV) (Ti—O) bonds, respectively.^44,45^ The Ti 2p core level spectra of MH and HMH3 display characteristics similar to those of pure MXene. Nevertheless, the Ti 2p_3/2_ peaks corresponding to Ti—C and Ti (II) in both MH and HMH3 shift toward higher binding energies by ∼0.50 and 0.45 eV, respectively (Table S1), indicating a stronger interaction of the Ti—C bonds and Ti atoms in the hydrogels. This enhanced interaction can be attributed to the strong association of the MXene nanosheets in the hydrogel structure.^37^ The enhanced interaction of the MXene nanosheets in MH and HMH3 is further evidenced by the shift of the C—Ti bond by ∼0.5 eV, as observed in the high-resolution C 1s XPS spectra (Figure 3e and Table S2). The significantly diminished C=O bond in the hydrogels as compared to pristine MXene reveals certain surface changes of MXene nanosheets upon gelation (Table S2). In addition, no significant increase in the TiO_2_ content was observed in HMH3 (8.4 wt %) compared to MH (9.4 wt %), as calculated from the Ti 2p XPS peak fitting, eliminating the possibility of extensive oxidation of MXene nanosheets during the etching treatment and formation of holey-MXene. As a result of this nominal oxidation, both MXene and holey-MXene nanosheets retain high electrical conductivities in MH and HMH3, respectively. Consequently, the interconnected highly conductive MXene and holey-MXene networks in MH and HMH3 endow the hydrogels with excellent electron conductivities, reaching up to 83.1 and 90.0 S cm^–1^, respectively (Figure 3f).

### Understanding the Assembly Mechanism and Formation of Holey-MXene Hydrogel

In order to elucidate the importance of [Zn(NH_3_)_4_]^2+^ cations in the formation of the stable and freestanding MXene hydrogel, we investigated the assembly process of MXene using a different conventional divalent cation, here the Zn^2+^ in aqueous ZnCl_2_ solution, as a gelation reagent. It is known that the basal plane of Ti_3_C_2_T_*x*_ MXene is decorated with polar functional groups (e.g., −OH, −O, and −F) stemming from the wet chemical etching process, which can anionize colloidal MXene in aqueous dispersion (a zeta potential of −39.7 mV).^46,47^ When divalent cations are introduced into the MXene dispersion, they interact electrostatically with the negatively charged MXene nanosheets, partially screening the electrostatic repulsion among the nanosheets and, thus, destabilizing the colloidal system. As a result, a transformation from the colloidal MXene dispersion to a cross-linked hydrogel occurs, with a decrease in the zeta potential value from −39.7 mV in MXene to −32.1 mV in the hydrogel.^48^ It was observed that, when only the aqueous ZnCl_2_ solution was employed to produce hydrogel, the as-formed hydrogel presented inferior structural stability as compared to the hydrogel formed by [Zn(NH_3_)_4_](OH)_2_, as evidenced by the rheology measurements (Figure S9a,b). Additionally, the morphological analysis of the MXene hydrogel formed by only ZnCl_2_ (Figure S10a,b) reveals severe restacking and agglomeration of MXene nanosheets in the hydrogel.

The possible explanation behind this observation is that hydrated Zn^2+^ cations in the aqueous ZnCl_2_ solution interact electrostatically with MXene nanosheets more strongly than the [Zn(NH_3_)_4_]^2+^ complex cations, that are larger in size and more screened by the four ammonia (NH_3_) ligands. A stronger electrostatic interaction offered by hydrated Zn^2+^ in the ZnCl_2_ solution rapidly breaks the repulsion among MXene nanosheets, resulting in more undesirable face-to-face restacking and agglomeration of the MXene nanosheets.^24^ Such agglomeration also prohibits the long-range interaction between the MXene nanosheets and cations, weakening the hydrogel structure. By contrast, the divalent complex cation [Zn(NH_3_)_4_]^2+^, formed by coordination of four NH_3_ ligands, exhibits a milder gelation effect toward MXene nanosheets and allows for better controlled cross-linking to attenuate the restacking and agglomeration behavior. The formation of this cross-linked structure is evident in the SEM images of freeze-dried MXene hydrogel (Figure S11a,b).

In the next step, 3 M aqueous H_2_SO_4_ solution was employed to wash out the [Zn(NH_3_)_4_]^2+^ cations at room temperature from the hydrogel. The removal of [Zn(NH_3_)_4_]^2+^ was confirmed by the absence of Zn species in the XPS survey spectra of MH (Figure S8). Notably, while washing out [Zn(NH_3_)_4_]^2+^ cations, the MXene sheets come close to each other and touch directly at the cross-linking points, which stabilizes the overall hydrogel strucutre. The comparison of the rheological analysis (Figure S12) reveals that the network of pure MXene nanosheets after removal of [Zn(NH_3_)_4_]^2+^ from MH is stronger than the [Zn(NH_3_)_4_]^2+^-cross-linked MXene nanosheets before acid-washing. As a result, MH prepared via acid-washing treatment presents higher *G*′ and *G*″ than their counterparts before acid-washing, indicating better mechanical robustness via first introducing and then removing [Zn(NH_3_)_4_]^2+^ cations from the hydrogel. Residual Li^+^ ions, used in the etching treatment of MAX, were not detected (Figure S8) and thus washed away as well along with [Zn(NH_3_)_4_]^2+^ cations during the acid wash, which also contributes to the enhanced mechanical properties of the MH hydrogel. The strong interaction among pure MXene nanosheets in MH is in accordance with the Raman and XPS results, as analyzed in the previous sections. Therefore, this multistep process including the gelation of MXene by [Zn(NH_3_)_4_]^2+^ and the subsequent acid-washing is capable of developing a freestanding, mechanically robust MXene hydrogel, which distinguishes itself from previously reported weak MXene hydrogels prepared via one-step rapid gelation induced by the hydrated cations alone.

The mechanism of the formation of nanoholes in HMHx by the mild oxidative etching process proceeds as follows. When MH is subjected to 3 M H_2_SO_4_ at 60 °C, MXene nanosheets are partially oxidized, and the oxidative products are *in situ* washed away by acid, leaving behind the nanoholes on the MXene surface.^33^ The oxidation is expected to primarily take place at the basal plane of the MXene nanosheets, particularly at the locations where there are defects or irregularities on the surface caused by the presence of surface terminal groups. As the MXene nanosheets undergo oxidation and etching, the functional groups present on the surface are partially removed and the defects caused by these groups gradually transform into nanoholes. The defect regions, where the surface terminal groups are present, are typically distributed throughout the basal plane of the MXene nanosheets. As a result, the etching process can proceed uniformly over the entire surface of the MXene nanosheets, resulting in evenly distributed in-plane nanoholes over the MXene surface, as observed through TEM studies (Figure 2f–h). As the etching process continues for a longer period, more nanoholes are created by the oxidation, and their size grows gradually (Figure 2f–h). This growth of the nanohole size is attributed to the increased oxidative reaction by H_2_SO_4_ around the edges of the defective pore regions in the MXene nanosheets.

### High-Rate Electrochemical Performance of HMHx Electrodes

The self-assembled freestanding HMHx with interconnecting porous structure, abundant in-plane nanoholes, and superior electrical conductivity is a promising electrode material for high-rate supercapacitors. To demonstrate their capacitive performance, electrochemical characterizations were first performed in a three-electrode Swagelok cell setup in an acidic electrolyte (3 M H_2_SO_4_). Meanwhile, the SEM image of the MXene electrode after Swagelok assembly shows a partial collapse of the large porous structure of the hydrogel (Figure S13a,b). However, the 3D open porous morphology of the electrode was not completely destroyed, and we can still observe channels and pores in the electrode structure following the Swagelok cell assembly, which are sufficient to maintain the diffusion of electrolyte ions.

**Figure 4:**
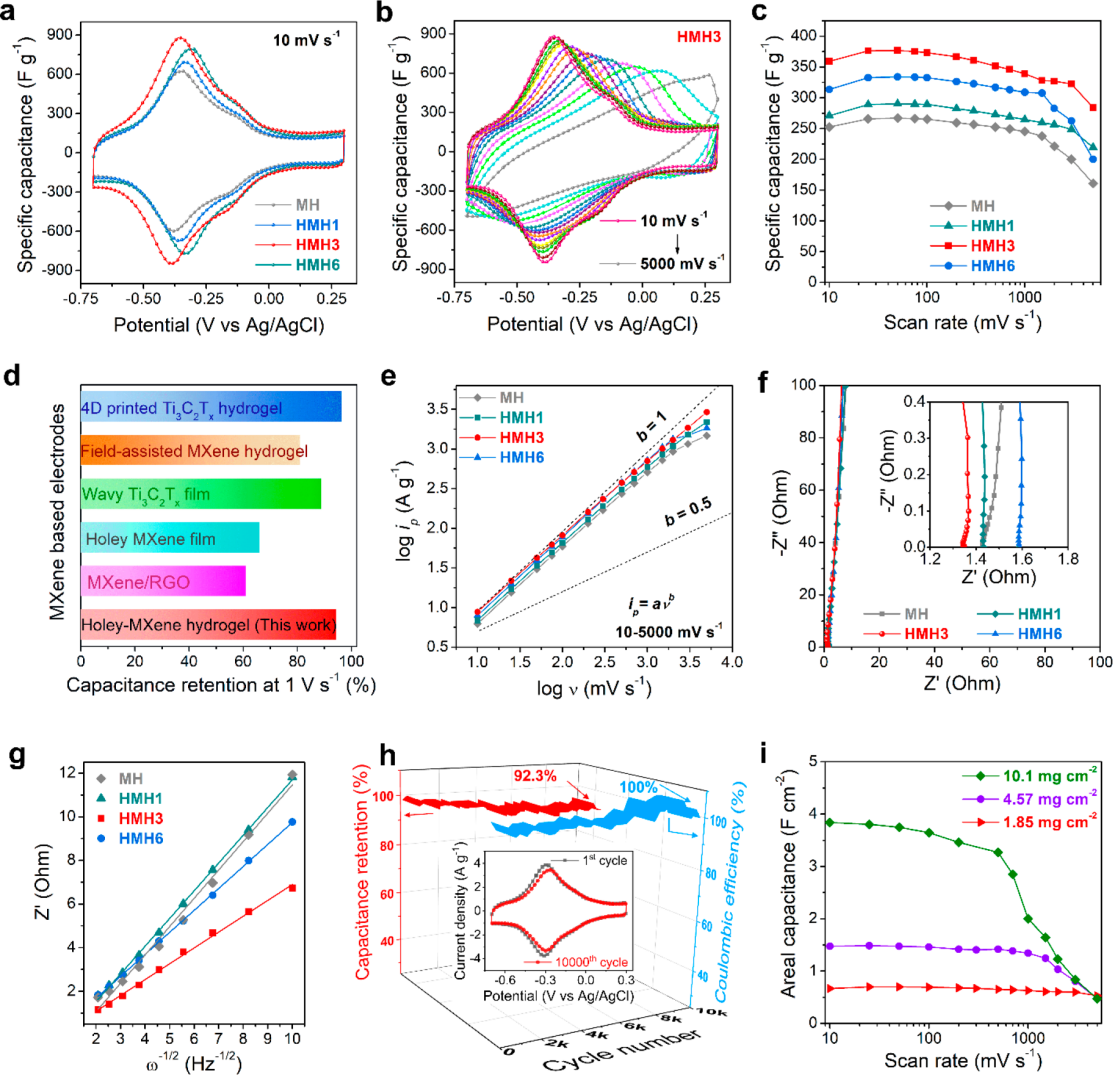
**Electrochemical characterizations of various MXene-hydrogel-based electrodes.** (a) CV profiles of MH and different HMHx electrodes at a scan rate of 10 mV s^–1^. (b) CV curves of HMH3 at scan rates of 10–5000 mV s^–1^. (c) Rate performance characteristics: variation of the specific capacitances of MH and different HMHx electrodes at scan rates of 10–5000 mV s^–1^. (d) Comparison of the specific capacitance of HMH3 with those of other reported MXene-based electrodes at 1000 mV s^–1^. (e) log *i*_*p*_ vs log *v* plot for different MXene-hydrogel-based electrodes for the determination of the *b* value. (f) Nyquist plot of different MXene-hydrogel-based electrodes. The inset shows a magnified view of the high-frequency region. (g) Variation of *Z*′ with ω^–1/2^ for different MXene-hydrogel-based electrodes. (h) Long-term stability test of the HMH3 electrode. The inset shows the CV curves of HMH3 at 10 mV s^–1^, before and after 10,000 GCD cycles. (i) Comparison of the areal capacitances of HMH3 electrodes at different mass loadings and scan rates.

As shown in the cyclic voltammetry (CV) profiles (Figure 4a), similar to pristine MH, all other HMHx, i.e., HMH1, HMH3, and HMH6, display distinct redox peaks within a potential range of −0.38 to −0.32 V vs Ag/AgCl reference electrode at a scan rate of 10 mV s^–1^. The presence of redox peaks in CV profiles indicates the pseudocapacitive charge-storage mechanism in the hydrogel electrodes originating from the inherent surface redox activities of MXene nanosheets.^49,50^ The galvanostatic charge–discharge (GCD) profiles of MH and HMHx (Figure S14a–d), which exhibit a slight deviation from the typical triangular character (for electrolytic double layer type capacitance (EDLC)), also confirm the pseudocapacitive behavior of these electrodes. These results further suggest that the holey-MXene nanosheets can preserve the intrinsic pseudocapacitive property of MXene. Importantly, the integral area of the CV profiles for the hydrogel electrodes increases significantly with the introduction of in-plane nanoholes on the MXene nanosheets, as shown in Figure 4a. The specific capacitance, calculated from the CV curves at a scan rate of 10 mV s^–1^, was measured to be 359.2, 313.5, 270.8, and 252.5 F g^–1^ for HMH3, HMH6, HMH1, and MH, respectively, representing better electrochemical performance of HMHx than MH. The improved capacitive performance of HMHx can be exclusively attributed to the presence of in-plane nanoholes, which substantially increase the number of electrochemically active reaction sites on MXene nanosheets.^51−53^

Figure 4b presents the CV profiles of the best-performing electrode, HMH3, at different scan rates ranging from 10 to 5000 mV s^–1^. Interestingly, only a negligible amount of distortion in the CV curves is observed at a high scan rate of 1000 mV s^–1^ with a small separation of cathodic and anodic peak potentials (<250 mV). HMH3 also carries a much lower value of the voltage drop (*iR*) (0.048 V at a high discharge current density of 384.6 A g^–1^) than MH (Figure S15), implying a lower equivalent series resistance (ESR) for HMH3. All of these results further validate the increased accessibility of holey-MXene nanosheets to electrolyte ions, which leads to a highly reversible and fast charge-storage process.

The quantitative analysis of the rate performance of various MXene-hydrogel-based electrodes is visualized in Figure 4c, which highlights the superior gravimetric specific capacitances (calculated from the CV profiles in Figure 4b and Figure S16a–c) of all HMHx at various scan rates in comparison to MH. In particular, the best-performing HMH3 exhibits specific capacitances of 339 and 283.9 F g^–1^ at ultrafast scan rates of 1000 and 5000 mV s^–1^, respectively. These values correspond to the respective capacitance retention of 94.4% and 79% of the initial capacitance at 10 mV s^–1^. By contrast, MH can maintain a specific capacitance of only 160.9 F g^–1^ at 5000 mV s^–1^, corresponding to 63.7% of its capacitance at 10 mV s^–1^. The significantly improved specific capacitance and the rate performance of the HMH3 electrode in comparison to MH clearly indicate the importance of the holey-MXene networks in the hydrogel structure. Figure 4d compares the specific capacitance and rate performance of the HMH3 electrode at 1000 mV s^–1^ with other reported MXene-based electrodes.^23,37,54−57^ It is evident that the specific capacitance of HMH3 at 1000 mV s^–1^ with a capacitance retention of 94.4% is one of the best electrodes ever reported so far and surpasses the performance of most of the state-of-art MXene-based electrodes (Table S3).

To comprehend the ultrafast charge-storage mechanism of HMH3, we analyzed the charge-storage kinetics of the electrode. It is known that the peak current densities (*i*_*p*_) of the CV curves at different scan rates (*v*) follow a power-law relationship: *i*_*p*_ = *av*^*b*^, where *a* and *b* are two variables.^58^ The value of *b* which can be determined from the slope of the log *i*_*p*_ vs log *v* plot provides important information about the charge-storage kinetics of the electrode. From Figure 4e, it is clear that HMH3 has a *b* value of 0.93, which is very close to 1, indicating that the charge-storage kinetics of the HMH3 electrode is dominated by the surface-controlled capacitive process. This fast surface capacitive process consists of EDLC as well as fast redox reactions originating from the surface pseudocapacitance of MXene nanosheets and accounts for the ultrafast charge-storage and excellent rate performance of the HMH3 electrode. In Figure 4e, it is evident that the other HMHx electrodes along with MH can also maintain a *b* value close to 1 over a wide range of scan rates, demonstrating the predominance of surface-controlled charge-storage kinetics in these electrodes. Nevertheless, a slight deviation of the *b* value for HMH6 and MH at high scan rates shows that the diffusion-limited process (*b* = 0.5) comes into play. This phenomenon can be ascribed to the increased restacking of the MXene nanosheets in HMH6 due to prolonged heating treatment (as evidenced by the XRD result in Figure S17), and also long and obstructed ion diffusion channels in MH owing to the absence of a holey-MXene network. Moreover, the prolonged heating may also lead to a decrease in the electronic conductivity in HMH6, adversely affecting the electrochemical performance of the electrode.

To further probe the charge-storage behavior of the hydrogel electrodes, electrochemical impedance spectroscopy (EIS) analysis was carried out, which can provide deep insights into the ion diffusion characteristics and charge transport kinetics of the electrodes. The Nyquist plots (Figure 4f), recorded over the frequency range of 100 kHz to 10 mHz, feature quasi-vertical lines in the low-frequency regime for all hydrogel electrodes, describing the capacitive behavior of these electrodes. A close-up investigation of the high-frequency region in the Nyquist plot (inset in Figure 4f) demonstrates the absence of a semicircular region, illustrating ultrafast charge-transfer characteristics. However, a small Warburg region (45° inclination) can be observed for MH in comparison to little to no Warburg regions for all the HMHx electrodes. This implies low diffusion resistances in the HMHx electrodes compared to MH owing to the rapid mass transport through the hierarchically porous architecture of HMHx. Moreover, the series resistance calculated from the Nyquist plots was found to be less than 1.6 Ω for all the hydrogel electrodes, indicating ultrafast charge transport through the conducting network of the MXene hydrogels.

Through the analysis of the Warburg impedance, we can gain more insights into the diffusion resistance and mass transport characteristics of the hydrogel electrodes. The Warburg factor (σ), which represents the Warburg impedance, is related to the diffusion coefficient of the electrolyte ions and the real part of the complex impedance (*Z*′) via the following equation: *Z*′ = *R*_*e*_ + *R*_*ct*_ + *σω*^–1/2^.^59^ Here, *R*_*e*_, *R*_*ct*_, and ω represent the equivalent resistance, charge transfer resistance, and angular frequency, respectively. Importantly, the slope of the linear fit of the plot *Z*′ vs ω^–1/2^, which represents σ, was found to be the smallest for HMH3 in comparison to the other HMHx and MH (Figure 4g). It indicates that HMH3 exhibits sufficiently low ion diffusion and charge-transfer resistances owing to the adequate and suitable nanohole distribution on the holey-MXene framework, which not only contributes to the fast and favorable ion diffusion kinetics but also maintains the conductivity of the hydrogel similar to pristine MXene hydrogel (Figure 3f). This observation is consistent with the findings obtained from the kinetics analysis and Nyquist plots, and corroborates the advantageous characteristics of HMHx electrodes, especially HMH3. The excellent performance of the HMH3 electrode is also reflected by its long-term stability test, as displayed in Figure 4h and the inset. HMH3 can retain 92.3% of its initial capacitance with an outstanding Coulombic efficiency of 100% for over 10,000 cycles of continuous charge–discharge at a high current density of 25 A g^–1^.

Apart from achieving a high specific capacitance, ensuring a high areal capacitance is crucial for a realistic supercapacitor system.^60^ This is because increasing areal capacitance within the limited footprint of the device ensures high cell-level energy and power densities.^61^ To investigate the effect of the mass loading on the areal capacitance of the best-performing HMH3 electrode, we systematically increased the mass loading of the electrode up to a commercial standard (∼10 mg cm^–2^). As depicted in Figure 4i, the HMH3 electrodes demonstrate outstanding areal specific capacitances, reaching values of 1.48 and 3.84 F cm^–2^ at the mass loadings of 4.57 and 10.1 mg cm^–2^, respectively, when tested at a scan rate of 10 mV s^–1^ (calculated from the CV curves shown in Figure S18a,b). In addition, HMH3 electrodes with mass loadings of 1.85 and 4.57 mg cm^–2^ can maintain the areal specifc capacitance values up to an ultrafast scan rate of 5000 mV s^–1^. Impressively, HMH3 with a commercial level of mass loading of 10.1 mg cm^–2^ does not exhibit significant decay in the areal specific capacitance up to a scan rate of 500 mV s^–1^ (>85% of capacitance retention). As indicated by the Nyquist plot (Figure S19 and inset), the charge transfer resistance in the 10.1 mg cm^–2^ electrode was found to increase slightly by 0.07 Ω due to the increased thickness compared with other HMH3 electrodes with relatively lower mass loadings. However, the diffusion resistance of the electrolyte did not show any noticeable increment for the 10.1 mg cm^–2^ electrode (inset in Figure S19), because of its hierarchically porous structure. As a result, the 10.1 mg cm^–2^ electrode can stably deliver an excellent areal specific capacitance of 2 F cm^–2^ at 1000 mV s^–1^ and 0.47 F cm^–2^ at 5000 mV s^–1^, which are significantly higher than the other high mass loading electrodes reported so far (Table S4). These findings further support the importance of the holey-MXene nanosheets in the HMH3 electrode, where the 3D hierarchically porous and conducting architecture favors rapid ion diffusion within the electrode, resulting in an exceptional areal capacitance, when subjected to a commercial-level mass loading in the electrode.

It is important to mention that 3D hydrogels have the limitation of low volumetric capacitance due to their low densities. In an effort to address the need for larger volumetric capacitance, we first disassembled HMH3 by ultrasonication and subsequently vacuum-filtered (Celgard 3501) the HMH3 dispersion. This process resulted in a thin film of HMH3 with a width of ∼10 μm, as illustrated in the SEM image in Figure S20a. The as-developed HMH3 film exhibited an ultrahigh volumetric capacitance of 1606.8 F cm^–3^ at a scan rate of 10 mV s^–1^. Moreover, this electrode retains 67.1% of the capacitance at a scan rate of 1000 mV s^–1^, demonstrating its outstanding rate performance (Figure S20b,c). The excellent volumetric capacitance and rate performance of the HMH3 film can be attributed to the compact layer-by-layer structure of the holey-MXene nanosheets, which ensures the fast electrolyte diffusion and reaction kinetics even in the thin film (as also evident from the Nyquist plot of the HMH3 film in Figure S20d).

It is worth noting that, while the three-electrode configuration is indispensable for characterizing the electrode, the performance evaluation of a supercapacitor in a symmetric cell setup, which represents the realistic device configuration, is important for practical applications.^62^ For this study, we tested the electrochemical performance of MH and the best-performing HMH3 in a symmetric two-electrode cell setup in 3 M H_2_SO_4_ electrolyte. The CV curves of both MH and HMH3 (Figure S21a,b) show a weak pair of redox peaks, especially at the slow scan rates, supporting the pseudocapacitive property of MXene. A comparison of the CV curves in Figure S21a,b reveals an enhanced electrochemical performance for HMH3 than MH. In addition, the CV curves of HMH3 (Figure S21b) can highly maintain its quasi rectangular shape up to a scan rate of 500 mV s^–1^, suggesting an impressive rate capability due to the efficient electrolyte ion transport in the electrode. The near vertical EIS spectrum of HMH3 (Figure S22 and inset), observed in the high-frequency regime, indicates the enhanced ion transport and reduced charge transfer resistance in HMH3 compared to the MH electrode. The specific gravimetric capacitance values, calculated from the CV curves (Figure S21a,b), were found to be 82 and 77 F g^–1^ for HMH3 and MH, respectively, at a scan rate of 10 mV s^–1^. More importantly, HMH3 displays an outstanding specific gravimetric capacitance of 33.1 F g^–1^ at an ultrafast scan rate of 5000 mV s^–1^, which corresponds to a rate capacitance of 40.4% (Figure S23). The values of the specific capacitances of HMH3 with superb rate performances are not only higher than MH but also surpass the performance of other MXene based symmetric capacitors reported so far.^55,63−65^ Besides, in this symmetric cell setup, the HMH3 electrode can retain 75% of its capacitance with a Coulombic efficiency of 95% for over 10,000 charge–discharge cycles at a high current density of 12.5 A g^–1^ (Figure S24). These results illustrate that the HMH3 electrode retains its excellent performance in the symmetric configuration, highlighting its potential for practical applications. Importantly, the Ragone plot (Figure S25) shows that, in the symmetric device configuration, HMH3 can deliver an ultrahigh energy density of 11.4 Wh kg^–1^ at a high power density of 410 W kg^–1^. Moreover, the HMH3 electrode is able to maintain a high energy density of 4.6 Wh kg^–1^ at an ultrahigh power density of 82.8 kW kg^–1^, which makes it more compelling in the practical scenario requiring a high energy density at a fast power output (Table S5). It is worth mentioning that aqueous electrolytes have the limitation of a low voltage window. The fabrication of hybrid devices, such as asymmetric supercapacitors or the use of organic or ionic liquids as electrolytes, can further enhance the energy density of the device.^66^

## Conclusions

Our studies showcased a facile strategy to develop freestanding, mechanically robust hydrogels of holey-MXene frameworks. In our concept, the mild electrostatic interaction of [Zn(NH_3_)_4_]^2+^ with negatively charged MXene nanosheets initiates a smooth cross-linking process among the MXene nanosheets. The multimode interactions involved in this synthesis, namely, interactions between MXene and [Zn(NH_3_)_4_]^2+^ during gelation and the subsequent interactions among pure MXene nanosheets after acid-washing to remove [Zn(NH_3_)_4_]^2+^, are believed as key factors in developing the freestanding hydrogel structure. Subsequently, a controlled oxidative acid-etching process forms abundant nanoholes on the surface of MXene nanosheets in the hydrogels. Our holey-MXene hydrogels with tunable nanohole size, enabled by etching time, displayed excellent specific capacitance and superb rate performance of 79% at 5000 mV s^–1^, when serving as supercapacitor electrodes. Besides, the electrode maintained a high capacitance retention of 52% at a scan rate of 1000 mV s^–1^, at the commercial standard mass loading. In the device configuration, the holey-MXene hydrogel can deliver an ultrahigh energy density of 11.4 Wh kg^–1^ at a power density of 410 W kg^–1^. We conclude that the interconnected porous framework of conducting holey-MXene nanosheets, featuring tunable in-plane nanoholes, enables efficient electron transport pathways and ion diffusion channels in three dimensions, amplifying the rate performance and energy storage capacity. This work demonstrates that the rational design of the MXene hydrogel electrode architecture can significantly enhance their energy storage performance, thereby advancing the potential applications of MXenes in commercial energy storage systems.

## Experimental Section

### Synthesis of Ti_3_C_2_T_*x*_ MXene Colloidal Dispersion

Ti_3_C_2_T_*x*_ MXene colloidal dispersion was prepared following a previously reported method.^32^ Briefly, Ti_3_AlC_2_ MAX powder (1 g) was slowly (over the course of 15 min) added into the mixture of LiF (1 g) and HCl (20 mL, 9 M), and was stirred continuously for 24 h at 35 °C. The as-obtained multilayered Ti_3_C_2_T_*x*_ dispersion was washed several times with deionized (DI) water through centrifugation and ultrasonicated for 1 h under argon purging to delaminate Ti_3_C_2_T_*x*_ layers. Finally the Ti_3_C_2_T_*x*_ dispersion was centrifuged again at 3500 rpm for 1 h, and the dark green supernatant of delaminated Ti_3_C_2_T_*x*_ sheets was collected as a colloidal MXene dispersion.

### Development of MXene Hydrogel

To prepare Ti_3_C_2_T_*x*_-based freestanding MXene hydrogel, first the divalent tetraamminezinc(II) complex cation ([Zn(NH_3_)_4_]^2+^) was synthesized. In a typical synthesis route, an aqueous solution of NaOH (0.2 mmol in 10 mL of water) was added directly to an aqueous solution of Zn(NO_3_)_2_·6H_2_O (0.1 mmol in 10 mL water) with continuous shaking, and the reaction was allowed to proceed at room temperature for 2 h.^67^ The resulting milky white precipitate was collected and washed several times with DI water by centrifugation. Subsequently, aqueous ammonia (NH_4_OH, 35%) was added to the precipitate, while continuously shaking until the dispersion became colorless (∼4 mL of NH_4_OH is required), indicating the formation of [Zn(NH_3_)_4_](OH)_2_ in water.

The as-formed [Zn(NH_3_)_4_](OH)_2_ was used as the gelation agent for developing a freestanding MXene hydrogel (MH). Briefly, an aqueous dispersion of MXene (1 mL, 10 mg mL^–1^) was added quickly to an aqueous solution of [Zn(NH_3_)_4_](OH)_2_ (200 μL) and the mixture was gently shaken a few times by hand. The gelation of MXene started to occur within a few seconds after the mixing, and the system was left undisturbed at room temperature for 2 h for further cross-linking. Finally, the resulting hydrogel was washed with H_2_SO_4_ (3 M) at ambient temperature to get rid of metal species and to obtain a freestanding pristine MXene hydrogel (MH). To develop MH with different shapes, different molds can be used, or the formed MH can also be cut into customized shapes. The size of the MH can easily be scaled up by proportionally increasing all of the precursor quantities. The hydrogel could be continuously washed with water to remove the acid. It could also be kept in H_2_SO_4_ (3 M) for electrochemical characterizations and the subsequent synthesis of holey-MXene hydrogel.

### Synthesis of Holey-MXene Hydrogel

To obtain holey-MXene hydrogels (HMHx, “x” stands for the duration of acid etching in hours), the as-developed MH, immersed in H_2_SO_4_ (5 mL, 3 M), was heated in an oven at 60 °C for 3 h. To control the size and distribution of the in-plane nanoholes on the MXene sheets, the individual hydrogels were prepared by keeping at 60 °C for 1, 3, and 6 h separately and were labeled as HMH1, HMH3, and HMH6, respectively. The resulting HMHx were placed in fresh H_2_SO_4_ (3 M) and directly used for electrochemical characterizations. For other characterizations, HMHx were dialyzed in water until a constant pH value was achieved.

### Material Characterization

The compression testing was done on the freeze-dried aerogels (cylindrical samples with a diameter of ∼12 mm and a height of ∼10 mm) using a universal mechanical testing machine (Instron 5960, USA) with a 1 kN load cell. The Micro-CT imaging was performed on a Zeiss Versa XRM520. The tomographic data acquisition involved 1601 projections of 1024 × 1024 pixels with an exposure time of 0.5 s, acquired over 360° sample rotation with the X-ray source set to 80 kV and 7 W. The projection data were reconstructed, with the Zeiss Reconstructor software, to yield a 3D image volume with cubic voxels of 2 μm width and an internal cylindrical field of view of about 2 mm in diameter. Visualization was performed with the Dragonfly software v.2021.1.977 (ORS, Montreal Canada). The morphology of the MXene hydrogels was characterized using a JEOL JSM-7000F scanning electron microscope (SEM) and a JEOL-2100 transmission electron microscope (TEM) operated at an accelerating voltage of 200 kV. An energy dispersive X-ray spectroscopy (EDX) detector (Oxford Instruments) fitted with a SEM was used for EDX elemental mapping. The thermogravimetric (TG) analysis was conducted using a Discovery instrument (TA Instruments) by heating the hydrogels from room temperature to 150 °C at a heating rate of 10 °C min^–1^ in a nitrogen environment. The X-ray diffraction (XRD) data was collected in powder mode with Cu Kα radiation at a wavelength of 1.5406 Å on a Bruker D8 DISCOVER X-ray diffractometer. Raman spectra were recorded on a Horiba (LabRam HR 800) laser micro Raman spectrometer using a laser source of 532 nm. The chemical compositions of MXene and MXene hydrogels were analyzed by ESCALAB 250XI (Thermo Fisher) X-ray photoelectron spectroscopy (XPS) instrument using a monochromatic Al Kα X-ray source (*hv* = 1486.6 eV). The nitrogen absorption/desorption measurements were performed with a Micromeritics ASAP 2020. Before performing the measurements, all the freeze-dried aerogel samples were degassed at 80 °C overnight. The rheological property of the hydrogel was tested in an Anton Paar MCR301 rheometer. To measure the electrical conductivity (S m^–1^), the MXene hydrogel with the dimensions of 1 × 0.5 × 0.8 cm^3^ was sandwiched between two platinum foils. The conductivity of the hydrogel was determined from the slope of the linear sweep voltammetry (LSV) plots, obtained at a scan rate of 10 mV s^–1^ and within a potential window of −0.2 to 0.2 V using a Biologic VSP300 potentiostat. The zeta potentials of the MXene dispersion and hydrogel were measured using a ZETA SIZER Nano ZS instrument (Malvern, UK) at room temperature.

### Electrochemical Characterization

All of the electrochemical characterizations were performed at room temperature in a Swagelok cell using a Biologic VSP300 potentiostat. In all of the electrochemical experiments, MXene hydrogels were cut and directly put onto a glassy carbon electrode (3 mm diameter) without any electrode processing such as adding binders or additives. The glassy carbon electrodes served as the current collector in the Swagelok cell. Three-electrode tests were conducted using MXene hydrogel on glassy carbon as the working electrode, overcapacitive activated carbon (YP-50, Kuraray, Japan) as the counter electrode, and Ag/AgCl (in 1 M KCl) as the reference electrode. A schematic of the Swagelok cell and its components for three-electrode test is shown in Figure S26. For two-electrode tests, MXene hydrogels on glassy carbon electrodes served as both of the electrodes. 3 M H_2_SO_4_ and Celgard 3501 membrane were used as the electrolyte and separator, respectively, in all the electrochemical tests. The cyclic voltammetry (CV) and galvanostatic charge–discharge (GCD) tests were carried out within a potential window of −0.7 to 0.3 V vs Ag/AgCl reference electrode. The electrochemical impedance spectroscopy (EIS) tests were conducted at a potential amplitude of 10 mV in a frequency range of 100 kHz to 10 mHz.

### Calculations of Various Parameters

The specific capacitance (*C*_*s*_, F g^–1^) of a single electrode was calculated from the CV curves (obtained from three-electrode tests) by using the following equation:
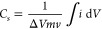
1In this equation, *i* represents the current (A) at a potential *V* (V), Δ*V* denotes the potential window (V) of CV scan, *v* is the scan rate (mV s^–1^), and *m* represents the mass (g) of the active material in the working electrode. The areal capacitance (*C*_*A*_, F cm^–2^) of a single electrode was calculated as follows
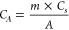
2where *A* is the area (cm^–2^) of the electrode.

For the two-electrode configuration, the device capacitance (*C*_*g*_, F g^–1^) was determined as
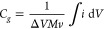
3where *M* is the total mass (g) of both electrodes.

The energy density (*E*_*g*_, Wh kg^–1^) and power density (*P*_*g*_, W kg^–1^) of the device were evaluated in the two-electrode configuration by using the following equations:
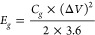
4
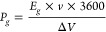
5

## Supporting Information Available

Additional information and figures: photographs of MXene dispersion, MH, and HMH3; TG analysis of MH and HMH3; TEM image of MH; SEM images of HMH1 and HMH6; N2 sorption isotherms and pore size distribution plots of MH and HMH3; XRD plots of MXene, MAX, and HMHx; XPS analysis of MXene, MH, and HMH3; detailed rheological analysis and SEM images of MH prepared by different cations; SEM images of MXene hydrogel electrodes (a) before and (b) after Swagelok assembly; charge–discharge curves, CV curves, MH, and HMHx; *iR* drop vs current density plots of MH and HMH3; CV curves and Nyquist plots of HMH3 at different electrode mass loadings; SEM image, CV curves, rate performance characteristics, and Nyquist plot of HMH3 film; CV curves, Nyquist plots, rate performance characteristics, long-term stability test, and Ragone plot of MH and HMH3 in symmetric electrode configuration; schematic of the Swagelok cell for three-electrode test; tables containing XPS peak assignments of MXene, MH, and HMH3; tables containing a comparison of the electrochemical performance of HMH3 with other reported supercapacitor electrodes (PDF)
