# Supplementary material for: Hierarchically Porous 3D Freestanding Holey-MXene Framework via Mild Oxidation of Self-Assembled MXene Hydrogel for Ultrafast Pseudocapacitive Energy Storage
Source: ACS Nano. 2024 Jan 17;18(4):3707–19. doi: 10.1021/acsnano.3c11551 (PMC10832346; doi:10.1021/acsnano.3c11551)
Supplement: Supplementary file 1 — nn3c11551_si_001.pdf [file nn3c11551_si_001.pdf]

Supporting Information

Hierarchically Porous 3D Freestanding Holey-MXene Framework *via* Mild Oxidation of Self-Assembled MXene Hydrogel for Ultrafast Pseudocapacitive Energy Storage

Anirban Sikdar[a], Frédéric Héraly[a], Hao Zhang[a], Stephen Hall[b], Kanglei Pang[a], Miao Zhang[a], Jiayin Yuan[a]**

[a]Department of Materials and Environmental Chemistry (MMK), Stockholm University, 10691 Stockholm, Sweden

[b]Division of Solid Mechanics, Lund University, 22100 Lund, Sweden

*Corresponding authors: jiayin.yuan@mmk.su.se (J. Yuan), miao.zhang@mmk.su.se (M. Zhang)

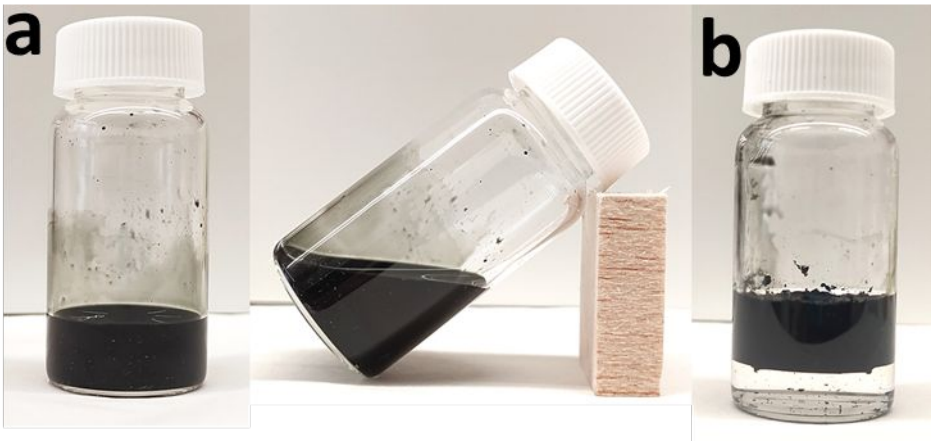

Figure S1. Photographs of a MXene dispersion and a MXene hydrogel. Digital images of a) MXene dispersion and b) as-formed MXene hydrogel (MH) obtained by mixing MXene with $[Zn(NH_3)_4]^{2+}$ cations.

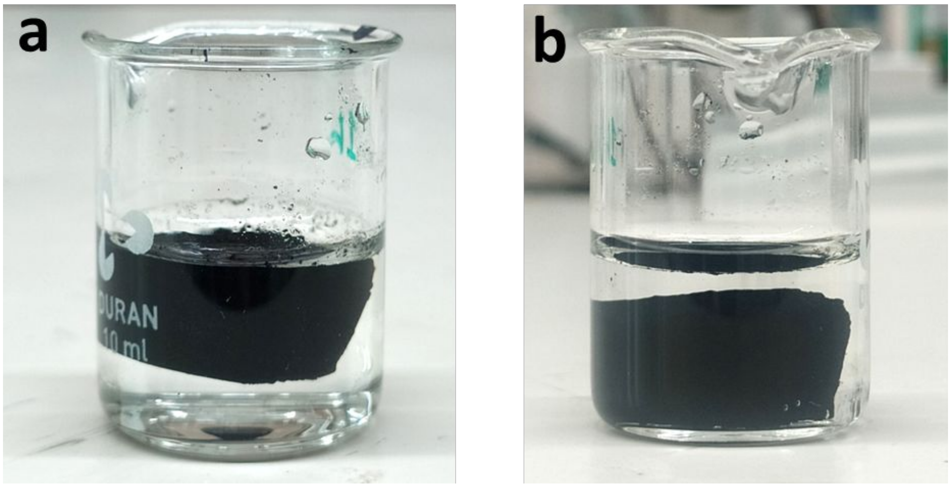

Figure S2. Photographs of MXene hydrogels. Digital images of a) acid washed MH and b) HMH3.

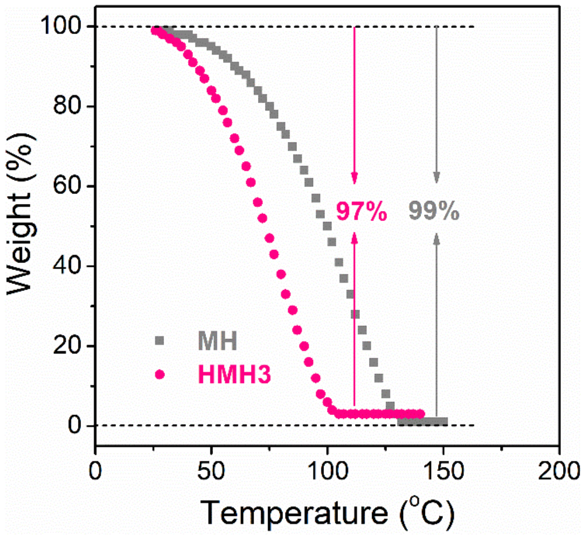

Figure S3. Characterization of MXene hydrogels. Thermogravimetric (TG) analysis of MH and HMH3, displaying the water content in the hydrogels.

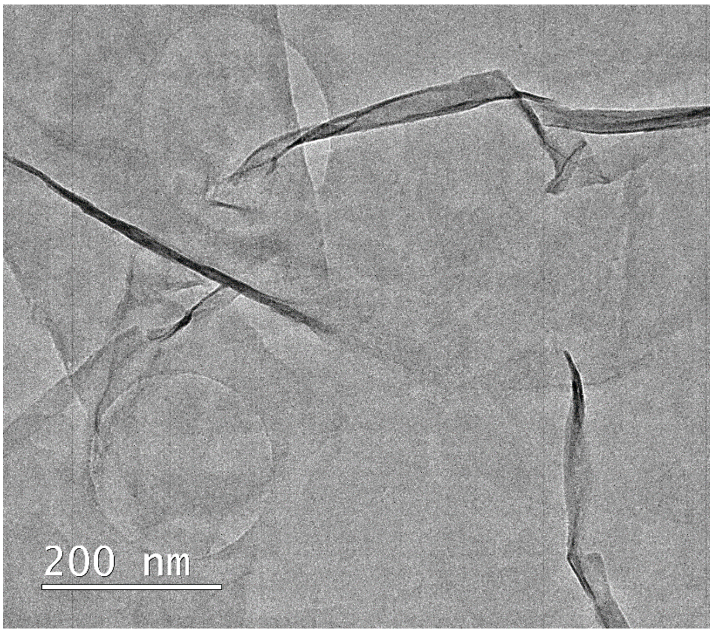

Figure S4. Morphological feature of MXene hydrogel. Low magnification TEM image of MH showing the clean surface feature and absence of any TiO_2 particles.

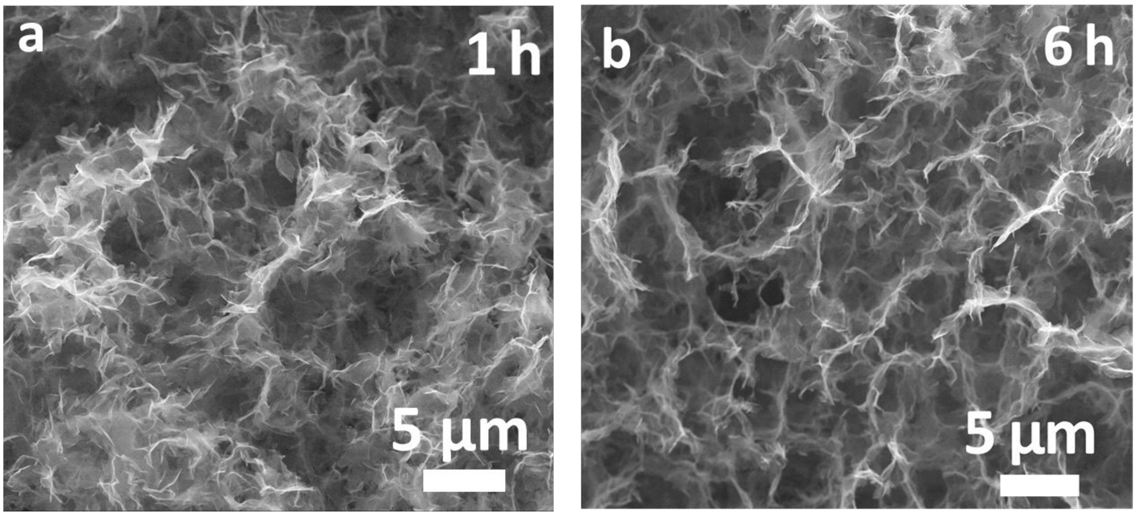

Figure S5. Morphological characterization of MXenes hydrogels. High magnification SEM images of a) HMH1 and b) HMH6 showing porous morphology of 3D hydrogel structure after freeze-drying, which confirms the mild etching process does not alter the microstructure of the MXene hydrogels.

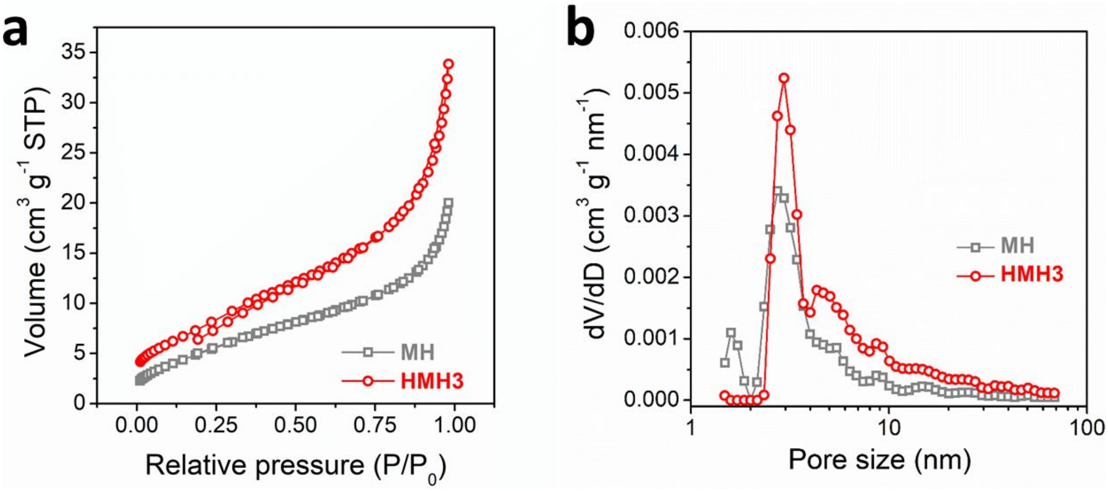

Figure S6. Determination of surface area and pore size distribution of MXene hydrogels. a) N_2 sorption isotherms, and b) pore size distribution plots of MH and HMH3.

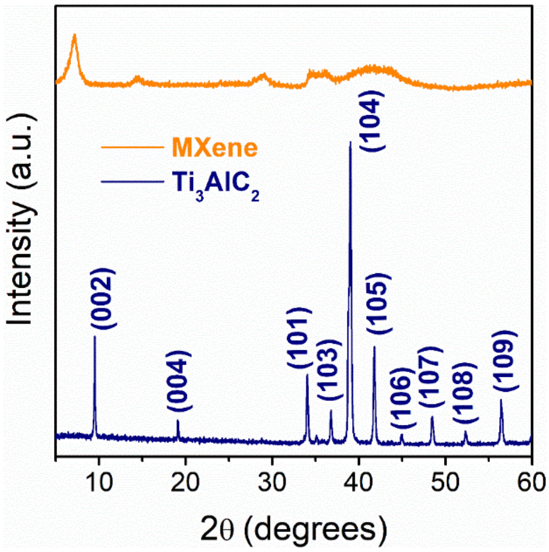

Figure S7. Phase characterization of MXene and MAX. XRD patterns of $Ti_3C_2T_x$ MXene and Ti_3AlC_2 MAX. The XRD peaks of the used MAX match well with the standard XRD pattern of Ti_3AlC_2 MAX.

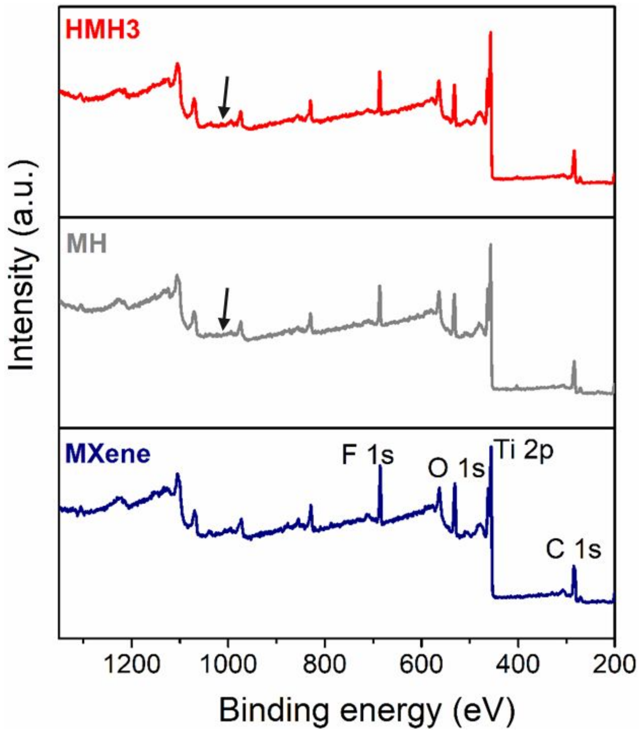

Figure S8. XPS analysis of MXene and MXene hydrogels. Comparison of the XPS survey spectra of pure MXene, MH and HMH3. The arrows in the spectra represent the possible location of Zn 2p peak, if Zn was present in the sample.

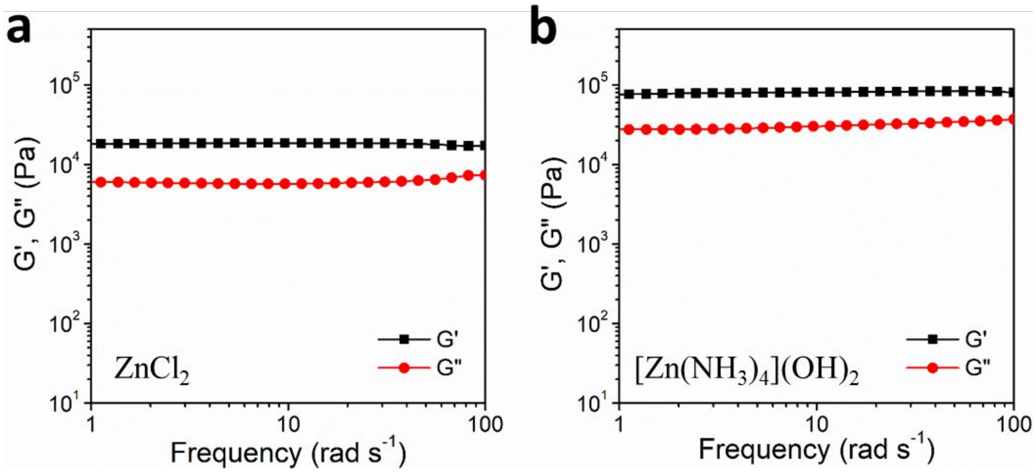

Figure S9. Rheological characterization of MXene hydrogels. Rheological characteristics of MXene hydrogels prepared using two Zn sources, (a) $ZnCl_2$ and (b) $[Zn(NH_3)_4](OH)_2$ before acid washing, displaying the typical hydrogel characteristics.

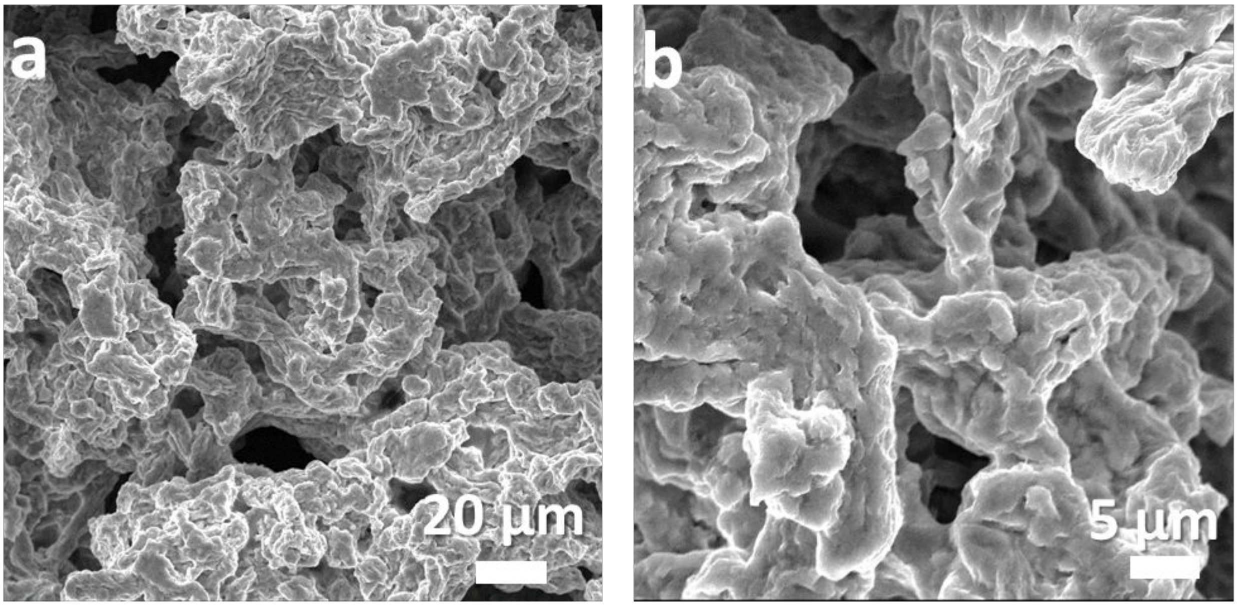

Figure S10. Morphological characterization of MXene hydrogels. (a) High and (b) low magnification SEM images of MXene hydrogel prepared by $ZnCl_2$ before washing with 3 M H_2SO_4, indicating agglomerated MXene sheets in the hydrogel structure.

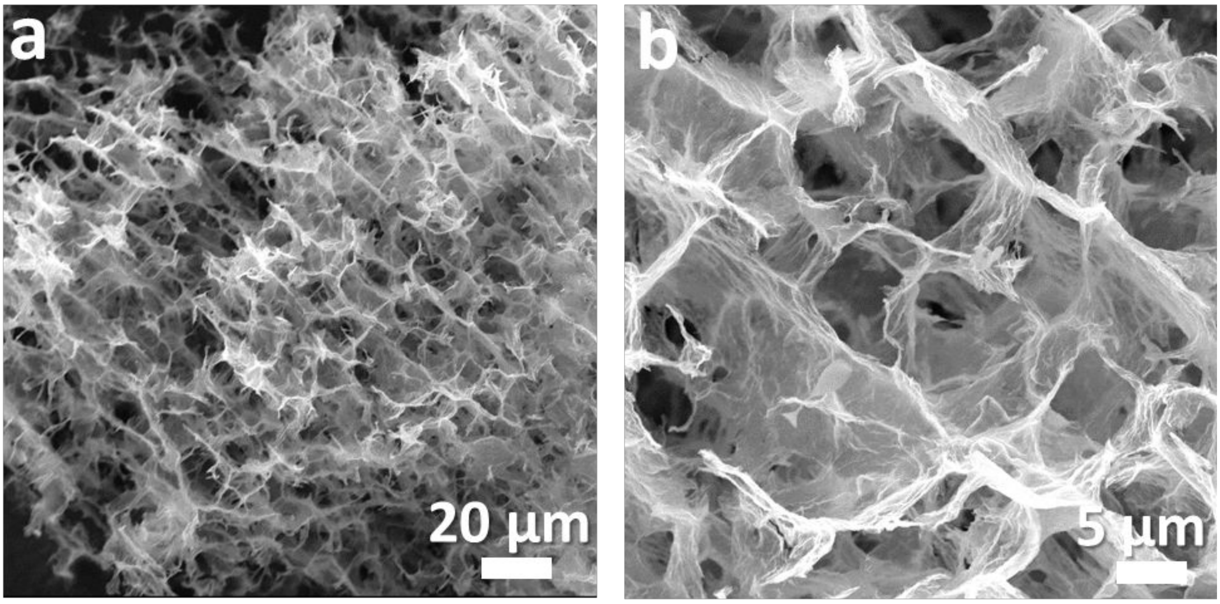

Figure S11. Morphological characterization of MXenes hydrogels. (a) High and (b) low magnification SEM images of MH (prepared using $[Zn(NH_3)_4]^{2+}$ cation) before washing with 3 M H_2SO_4, illustrating the formation of restacking-free and well-structured MXene hydrogel.

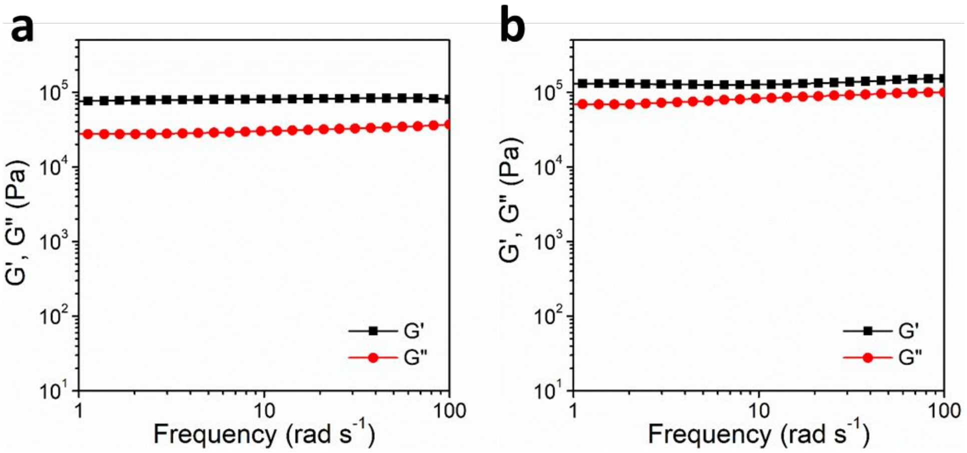

Figure S12. Rheological characterization of MH. Comparison of the rheological characteristics of MH, (a) before and (b) after acid wash, displaying the acid washing strengthens the hydrogel structure.

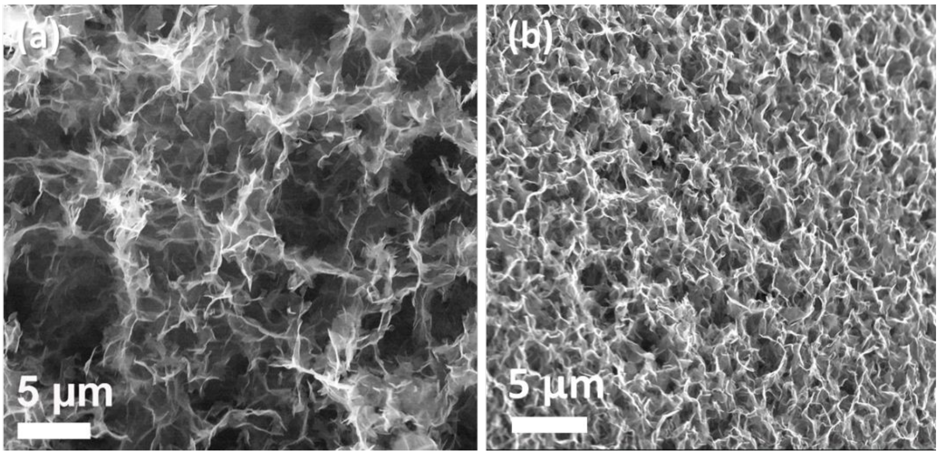

Figure S13. Morphology analysis of MXene electrode. SEM images of MXene hydrogel electrodes (a) before and (b) after Swagelok assembly.

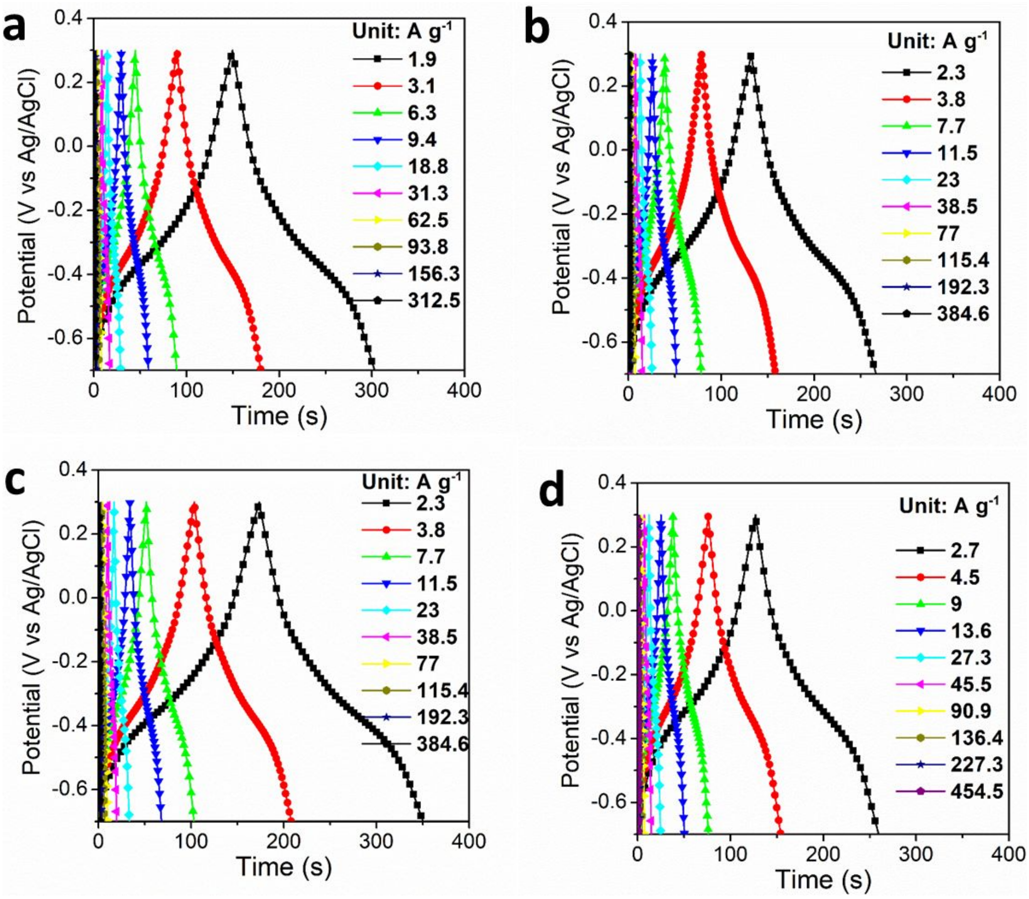

Figure S14. Electrochemical characterization of various MXene hydrogels. GCD curves of (a) MH, (b) HMH1, (c) HMH3, and (d) HMH6 at different current densities. The non-linear characteristics of GCD profiles indicate pseudocapacitive behavior of MXene.

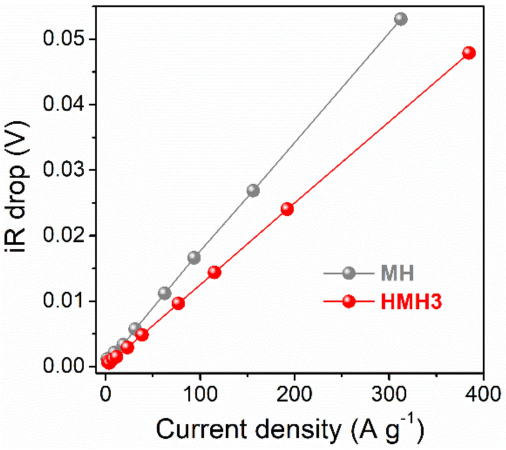

Figure S15. Resistive drop characterization of different MXene hydrogels. Comparison of the iR drop values of MH and HMH3 at different current densities calculated from their respective GCD curves.

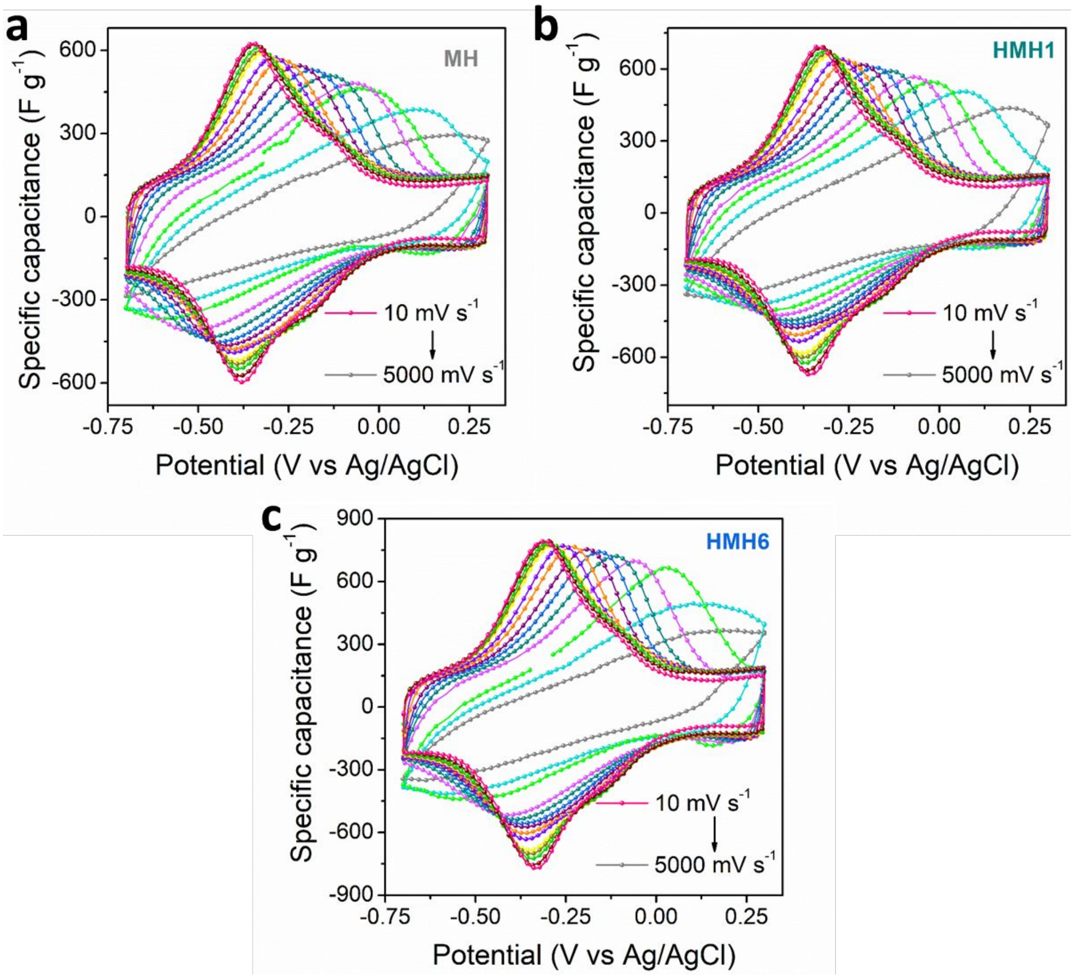

Figure S16. Electrochemical characterization of various MXene hydrogels. CV curves of (a) MH, (b) HMH1, and (c) HMH6 at different scan rates from 10 to 5000 mV s^{-1}.

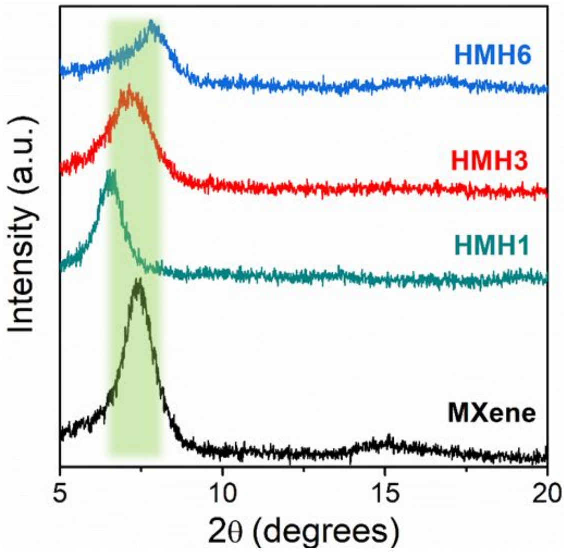

Figure S17. Phase characterization of MXene and various MXene hydrogels. Comparison of the XRD patterns of different holey-MXene hydrogels (HMH1, HMH3 and HMH6) and pristine MXene. Increase in etching time (from 1h in HMH1 to 6h in HMH6) causes minor restacking of MXene sheets in the hydrogel.

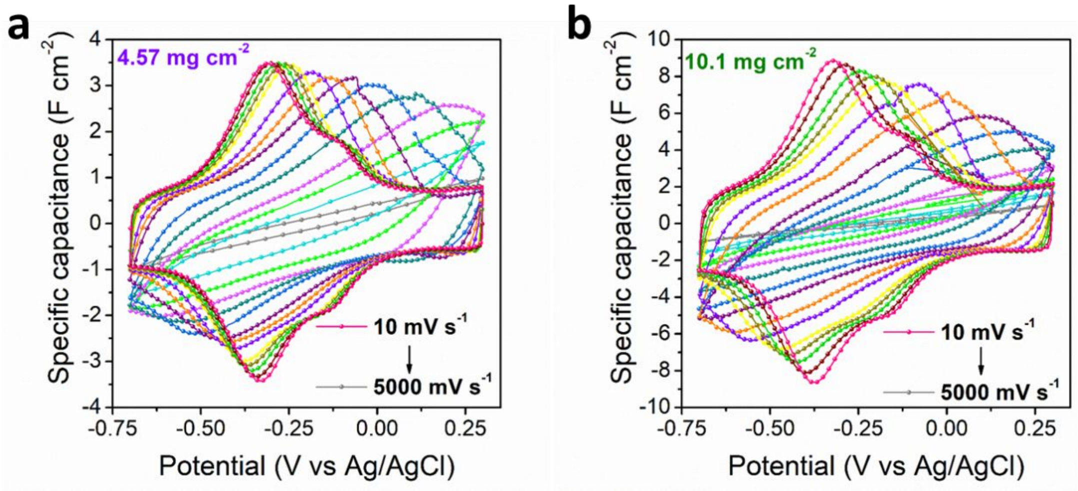

Figure S18. Electrochemical characterization of various MXene hydrogels. CV profiles of holey-MXene hydrogels with mass loadings at (a) 4.57 mg cm^{-2} and (b) 10.1 mg cm^{-2} at different scan rates.

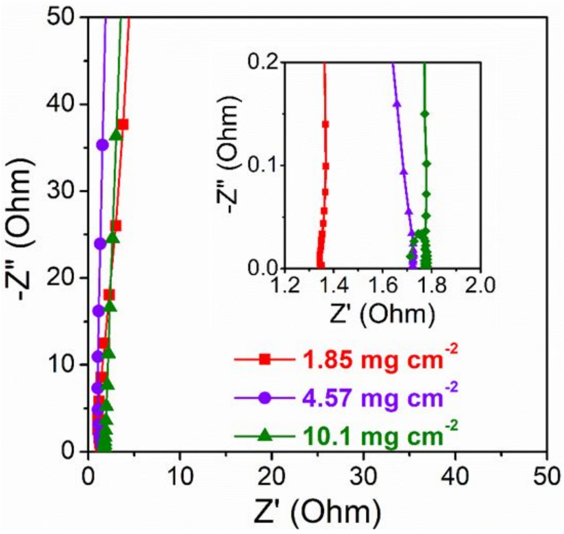

Figure S19. Electrochemical characterization of various MXene hydrogels. Comparison of the EIS spectra of holey-MXene hydrogels at different electrode mass loadings.

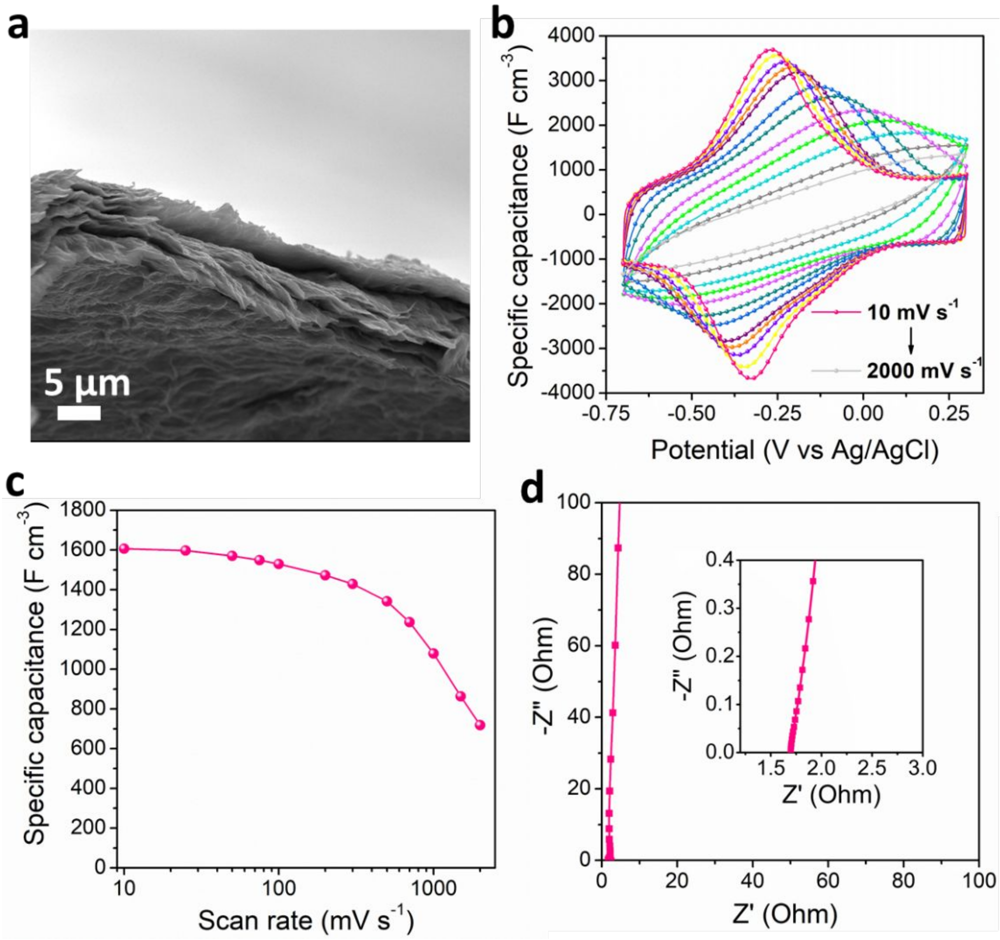

Figure S20. Characterization of HMH3 film. (a) Cross-sectional SEM image, (b) CV profiles at scan rates 10 - 2000 mV s^{-1}, (c) rate performance characteristics, and (d) EIS spectrum of

HMH3 film. The inset in (d) shows a zoomed view in EIS spectrum at the high frequency regime. The absence of charge-transfer characteristics and near zero Warburg region in (d) demonstrate the fast reaction kinetics and electrolyte diffusion in the HMH3 film electrode.

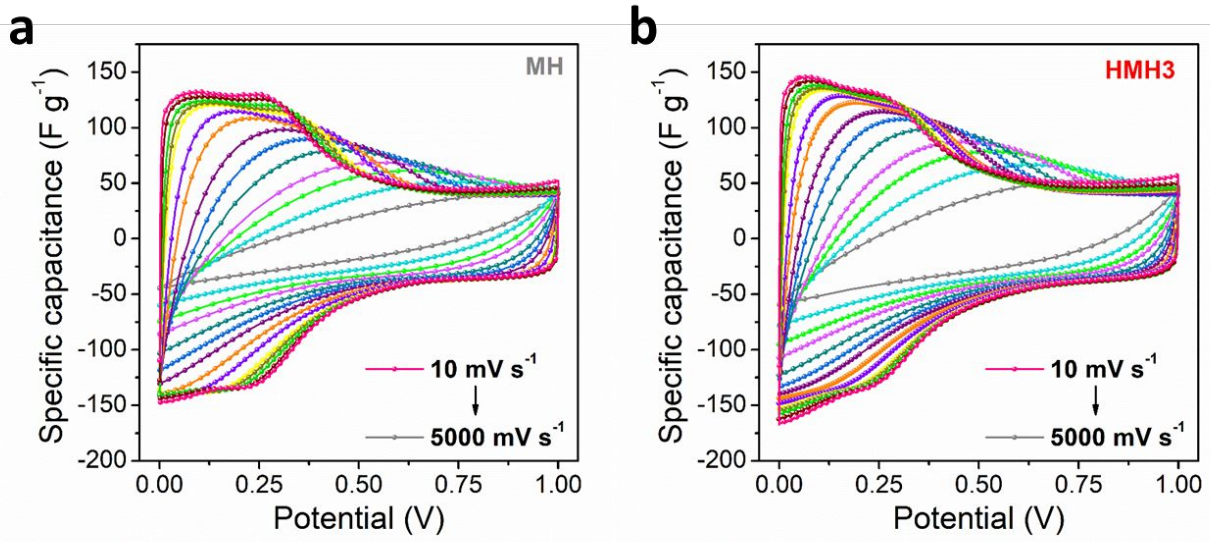

Figure S21. Electrochemical characterization of various MXene hydrogels. CV profiles of (a) MH and (b) HMH3 at different scan rates in symmetric two-electrode configuration.

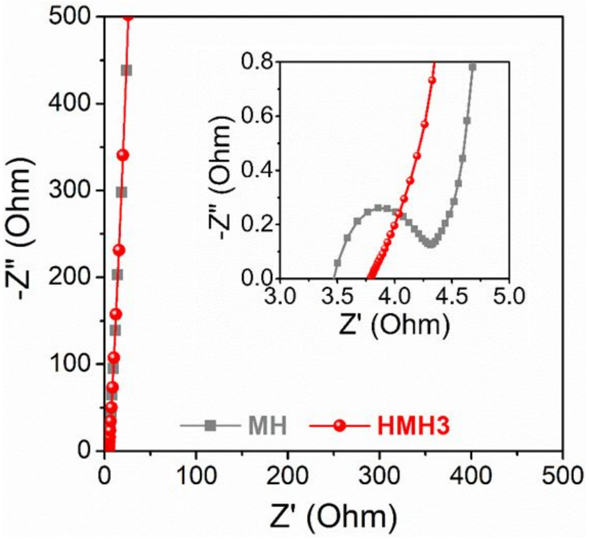

Figure S22. Electrochemical characterization of various MXene hydrogels. EIS spectra of (a) MH and (b) HMH3 in symmetric two-electrode configuration. The inset shows a zoomed view in EIS spectra at the high frequency regime. The absence of charge-transfer resistance in HMH3 indicates a significantly increased number of active-sites due to the introduction of nanoholes on the surface of MXene.

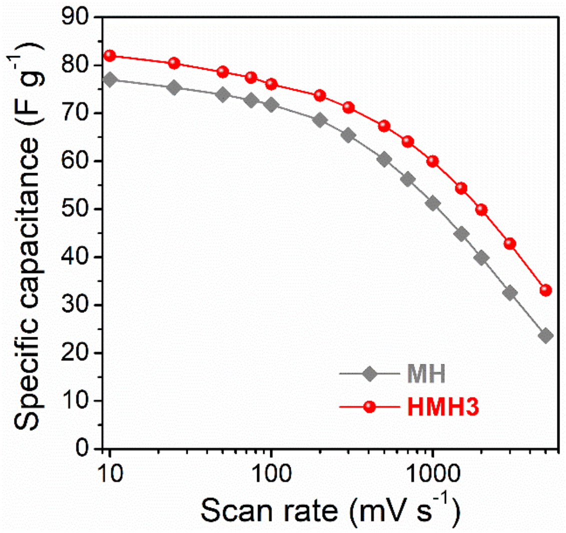

Figure S23. Rate performance characteristics. Variation of specific capacitance of MH and HMH3 at different scan rates in symmetric two-electrode configuration.

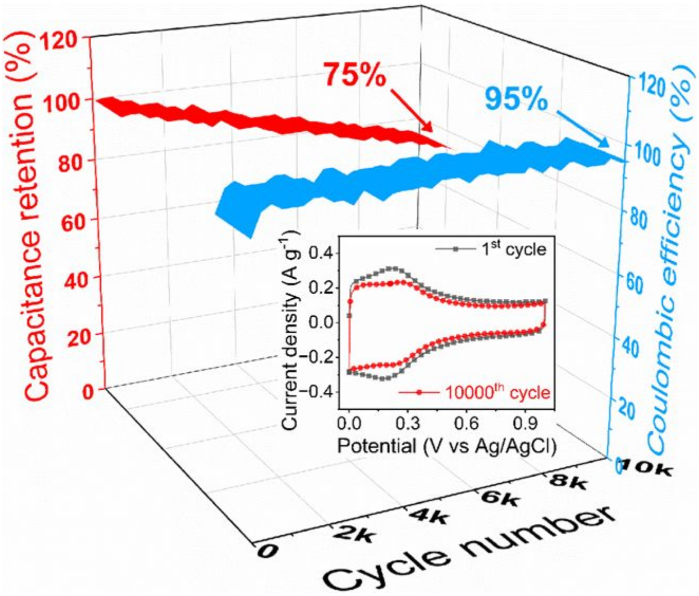

Figure S24. Long term stability test. Variation of capacitance retention and Coulombic efficiency with number of charge-discharge cycles for HMH3 in symmetric two-electrode configuration. The inset shows the comparison of CV curves before and after the stability test.

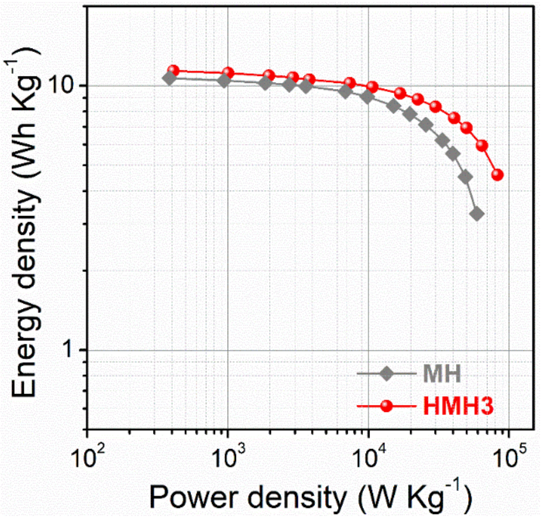

Figure S25. Supercapacitor performance test for various MXene hydrogels. Ragone plot of MH and HMH3 displaying the variation of energy densities and power densities of the devices at different scan rates.

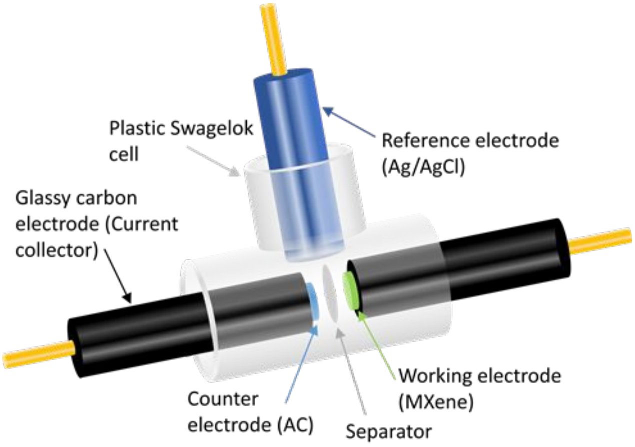

Figure S26. Three-electrode test. Schematic of the Swagelok cell for three-electrode test showing the cell configuration and its components.

Table S1. Assignments of Ti 2p XPS spectra of pure MXene, MH and HMH3

Assignment of Ti 2p core level spectra		MXene	MH	HMH3
Ti-C	$2p_{3/2}$	455.2 eV	455.72 eV	455.73 eV
	$2p_{1/2}$	461.38 eV	461.35 eV	461.1 eV
Ti (II)	$2p_{3/2}$	455.87 eV	456.29 eV	456.27 eV
	$2p_{1/2}$	461.59 eV	462.04 eV	462.06 eV
Ti (III)	$2p_{3/2}$	457.19 eV	457.45 eV	457.45 eV
	$2p_{1/2}$	462.7 eV	463.1 eV	463.06 eV
Ti (IV)	$2p_{3/2}$	458.99 eV	458.8 eV	459.21 eV
	$2p_{1/2}$	465.79 eV	464.28 eV	464.23 eV

Table S2. Assignments of C 1s XPS spectra of pure MXene, MH and HMH3

Assignment of C 1s core level spectra	MXene	MH	HMH3
C-Ti	282.2 eV	282.73 eV	282.75 eV
C-C	284.7 eV	284.8 eV	284.8 eV
C-O	286.2 eV	286.24 eV	286.5 eV
C=0	287.99 eV	–	–

Table S3. Comparison of the electrochemical performance of HMH3 with other reported supercapacitor electrodes

Electrode	Electrolyte	Mass loading	Specific capacitance	Rate performance	Reference
**Holey-MXene hydrogel (HMH3)**	**3 M H_2SO_4**	**1.85 mg cm^{-2}**	**359.2 F g^{-1} at 10 mV s^{-1}**	**79% at 5000 mV s^{-1}**	**This work**
d-Ti_3C_2 film	1 M Li_2SO_4	1.6 mg cm^{-2}	191.5 F g^{-1} at 2 mV s^{-1}	51% at 200 mV s^{-1}	[1]
MXene/rGO film	3 M H_2SO_4	-	335.4 F g^{-1} at 2 mV s^{-1}	61% at 1000 mV s^{-1}	[2]
$Ti_3C_2T_x$ clay	1 M H_2SO_4	-	245 F	80% at 100 mV s^{-1}	[3]
Wavy $Ti_3C_2T_x$ film	3 M H_2SO_4	2.28 mg cm^{-2}	299 F g^{-1} at 10 mV s^{-1}	69.5% at 5000 mV s^{-1}	[4]
Filtered $Ti_3C_2T_x$ hydrogel	3 M H_2SO_4	1.2 mg cm^{-2}	380 F g^{-1} at 2 mV s^{-1}	10% at 10000 mV s^{-1}	[5]

3D graphene/MXene hydrogel	3 M H_2SO_4	2.9 mg cm^{-2}	357 F g^{-1} at 10 mV s^{-1}	60% at 500 mV s^{-1}	[6]
$Ti_3C_2T_x$/rGO Hydrogel film	3 M H_2SO_4	1.16 mg cm^{-2}	294.5 F g^{-1} at 10 mV s^{-1}	75% at 2000 mV s^{-1}	[7]
$Ti_3C_2T_x$/NbN film	1 M H_2SO_4	1.05 mg cm^{-2}	277 F g^{-1} at 10 mV s^{-1}	50% at 2000 mV s^{-1}	[8]
$Ti_3C_2T_x$ -PVA	1 M KOH	-	167	56.8% at 100 mV s^{-1}	[9]
Porous 3D $Ti_3C_2T_x$ film	3 M H_2SO_4	0.53 mg cm^{-2}	354.8 F g^{-1} at 10 mV s^{-1}	58.6% at 10000 mV s^{-1}	[10]
N-doped $Ti_3C_2T_x$	1 M H_2SO_4	3-4 mg cm^{-2}	192 F g^{-1} at 1 mV s^{-1}	67% at 200 mV s^{-1}	[11]
MXene	1 M H_2SO_4	1.3 mg cm^{-2}	325 F g^{-1} at 10 mV s^{-1}	42.3% at 100 mV s^{-1}	[12]
$Ti_3C_2T_x$/MWCNT paper	1 M Mg_2SO_4	-	150 F g^{-1} at 2 mV s^{-1}	78% at 200 mV s^{-1}	[13]
MXene hydrogel	3 M H_2SO_4	-	272 F g^{-1} at 2 mV s^{-1}	83% at 1000 mV s^{-1}	[14]
Field-assisted MXene hydrogel	3 M H_2SO_4	0.5 mg cm^{-2}	395 F g^{-1} at 2 mV s^{-1}	42% at 5000 mV s^{-1}	[15]
4D-printed MXene hydrogel	3 M H_2SO_4	0.5 mg cm^{-2}	284 F g^{-1} at 10 mV s^{-1}	82% at 10000 mV s^{-1}	[16]
liquid-mediated holey MXene film	3 M H_2SO_4	0.5 mg cm^{-2}	360 F g^{-1} at 10 mV s^{-1}	66% at 1000 mV s^{-1}	[17]
Small sheet MXene hydrogel	3 M H_2SO_4	1.2 mg cm^{-2}	342.6 F g^{-1} at 2 mV s^{-1}	76% at 1000 mV s^{-1}	[18]
Rolled MXene aerogel	3 M H_2SO_4	0.6 mg cm^{-2}	315 F g^{-1} at 10 mV s^{-1}	39% at 200 mV s^{-1}	[19]
d-Mo_2CT_x film	1 M H_2SO_4	0.6 mg cm^{-2}	196 F g^{-1} at 2 mV s^{-1}	61.2% at 100 mV s^{-1}	[20]

Table S4. Comparison of areal capacitances and rate performances of various high mass loading electrodes

Electrode	Mass loading	Areal capacitance at high scan rate	Rate performance	Reference
**Holey-MXene hydrogel (HMH3)**	**10.1 mg cm^{-2}**	**2 F cm^{-2} at 1000 mV s^{-1} and 0.47 F cm^{-2} at 5000 mV s^{-1}**	**52% at 1000 mV s^{-1}**	**This work**
Filtered $Ti_3C_2T_x$ hydrogel	11.3 mg cm^{-2}	0.46 F cm^{-2} at 1000 mV s^{-1}	12.5% at 1000 mV s^{-1}	[5]
4D-printed MXene hydrogel	11.8 mg cm^{-2}	0.88 F cm^{-2} at 1000 mV s^{-1}	26.7% at 1000 mV s^{-1}	[16]
Small sheet MXene hydrogel	14.9 mg cm^{-2}	4.1 F cm^{-2} at 100 mV s^{-1}	82% at 100 mV s^{-1}	[18]
Porous MXene film	12 mg cm^{-2}	0.66 F cm^{-2} at 1000 mV s^{-1}	22% at 1000 mV s^{-1}	[21]

Ti_3C_2 aerogel	15 mg cm^{-2}	0.49 F cm^{-2} at 100 mV s^{-1}	47.9% at 100 mV s^{-1}	[22]
MXene-graphene hydrogel	9.2 mg cm^{-2}	1 F cm^{-2} at 100 mV s^{-1}	68% at 100 mV s^{-1}	[23]

Table S5. Comparison of the gravimetric energy and power densities of various MXene-based electrodes

Electrode	Highest energy density	Corresponding power density	Energy density at maximum power density	Reference
**HMH3**	**11.4 Wh kg^{-1}**	**410 W kg^{-1}**	**4.6 Wh kg^{-1} at 82800 W kg^{-1}**	**This work**
Macroporous MXene film	9.65 Wh kg^{-1}	45 W kg^{-1}	-	[24]
MXene-graphene hydrogel	10.23 Wh kg^{-1}	460.35 W kg^{-1}	4.65 Wh kg^{-1} at 10400 W kg^{-1}	[23]
Nanoporous MXene film	6.3 Wh kg^{-1}	56 W kg^{-1}	-	[25]
MXene/holey graphene	14.84 Wh kg^{-1}	250 W kg^{-1}	12.06 Wh kg^{-1} at 5000 W kg^{-1}	[26]
3D $Ti_3C_2T_x$/CNT film	9.2 Wh kg^{-1}	96.1 W kg^{-1}	2.8 Wh kg^{-1} at 15000 W kg^{-1}	[27]
rGO/MXene-PPy	11.3 Wh kg^{-1}	500 W kg^{-1}	5.6 Wh kg^{-1} at 4000 W kg^{-1}	[28]
3D graphene/ MXene hydrogel	9.3 Wh kg^{-1}	500 W kg^{-1}	5.7 Wh kg^{-1} at 5000 W kg^{-1}	[39]
freeze-dried MXene film	6.1 Wh kg^{-1}	175 W kg^{-1}	-	[30]
N-doped mesoporous carbon/MXene	8.1 Wh kg^{-1}	250 W kg^{-1}	2.5 Wh kg^{-1} at 12750 W kg^{-1}	[31]

References:

1. Yang, C.; Tang, Y.; Tian, Y.; Luo, Y.; He, Y.; Yin, X.Que, W. Achieving of Flexible, Free-Standing, Ultracompact Delaminated Titanium Carbide Films for High Volumetric Performance and Heat-Resistant Symmetric Supercapacitors. *Adv. Funct. Mater.* **2018**, 28, 1705487.

2. Yan, J.; Ren, C. E.; Maleski, K.; Hatter, C. B.; Anasori, B.; Urbankowski, P.; Sarycheva, A.Gogotsi, Y. Flexible MXene/Graphene Films for Ultrafast Supercapacitors with Outstanding Volumetric Capacitance. *Adv. Funct. Mater.* **2017**, 27, 1701264.

3. Ghidiu, M.; Lukatskaya, M. R.; Zhao, M.-Q.; Gogotsi, Y.Barsoum, M. W. Conductive two-dimensional titanium carbide 'clay' with high volumetric capacitance. *Nature*. **2014**, 516, 78-81.

4. Li, K.; Wang, X.; Wang, X.; Liang, M.; Nicolosi, V.; Xu, Y.Gogotsi, Y. All-pseudocapacitive asymmetric MXene-carbon-conducting polymer supercapacitors. *Nano Energy*. **2020**, 75, 104971.

5. Lukatskaya, M. R.; Kota, S.; Lin, Z.; Zhao, M.-Q.; Shpigel, N.; Levi, M. D.; Halim, J.; Taberna, P.-L.; Barsoum, M. W.; Simon, P.Gogotsi, Y. Ultra-high-rate pseudocapacitive energy storage in two-dimensional transition metal carbides. *Nat. Energy*. **2017**, 2, 17105.

6. Sikdar, A.; Dutta, P.; Deb, S. K.; Majumdar, A.; Padma, N.; Ghosh, S.Maiti, U. N. Spontaneous three-dimensional self-assembly of MXene and graphene for impressive energy and rate performance pseudocapacitors. *Electrochim. Acta*. **2021**, 391, 138959.

7. Wu, Z.; Liu, X.; Shang, T.; Deng, Y.; Wang, N.; Dong, X.; Zhao, J.; Chen, D.; Tao, Y.Yang, Q.-H. Reassembly of MXene Hydrogels into Flexible Films towards Compact and Ultrafast Supercapacitors. *Adv. Funct. Mater.* **2021**, 31, 2102874.

8. Wang, H.; Li, J.; Kuai, X.; Bu, L.; Gao, L.; Xiao, X.Gogotsi, Y. Enhanced Rate Capability of Ion-Accessible $Ti_3C_2T_x$-NbN Hybrid Electrodes. *Adv. Energy Mater.* **2020**, 10, 2001411.

9. Ling, Z.; Ren, C. E.; Zhao, M.-Q.; Yang, J.; Giammarco, J. M.; Qiu, J.; Barsoum, M. W.Gogotsi, Y. Flexible and conductive MXene films and nanocomposites with high capacitance. *Proc. Natl. Acad. Sci.* **2014**, 111, 16676-16681.

10. Kong, J.; Yang, H.; Guo, X.; Yang, S.; Huang, Z.; Lu, X.; Bo, Z.; Yan, J.; Cen, K.Ostrikov, K. K. High-Mass-Loading Porous Ti3C2Tx Films for Ultrahigh-Rate Pseudocapacitors. *ACS Energy Letters*. **2020**, 5, 2266-2274.

11. Wen, Y.; Rufford, T. E.; Chen, X.; Li, N.; Lyu, M.; Dai, L.Wang, L. Nitrogen-doped $Ti_3C_2T_x$ MXene electrodes for high-performance supercapacitors. *Nano Energy*. **2017**, 38, 368-376.

12. Dall'Agnese, Y.; Lukatskaya, M. R.; Cook, K. M.; Taberna, P.-L.; Gogotsi, Y.Simon, P. High capacitance of surface-modified 2D titanium carbide in acidic electrolyte. *Electrochem. Commun.* **2014**, 48, 118-122.

13. Zhao, M.-Q.; Ren, C. E.; Ling, Z.; Lukatskaya, M. R.; Zhang, C.; Van Aken, K. L.; Barsoum, M. W.Gogotsi, Y. Flexible MXene/Carbon Nanotube Composite Paper with High Volumetric Capacitance. *Adv. Mater.* **2015**, 27, 339-345.
14. Deng, Y.; Shang, T.; Wu, Z.; Tao, Y.; Luo, C.; Liang, J.; Han, D.; Lyu, R.; Qi, C.; Lv, W.; Kang, F.Yang, Q.-H. Fast Gelation of Ti3C2Tx MXene Initiated by Metal Ions. *Adv. Mater.* **2019**, 31, 1902432.
15. Dutta, P.; Deb, S. K.; Patra, A.; Majumdar, A.; Karim, G. M.; Parashar, C. K.; Mohanta, M. K.; Qureshi, M.Maiti, U. N. Electric Field Guided Fast and Oriented Assembly of MXene into Scalable Pristine Hydrogels for Customized Energy Storage and Water Evaporation Applications. *Adv. Funct. Mater.* **2022**, 32, 2204622.
16. Li, K.; Zhao, J.; Zhussupbekova, A.; Shuck, C. E.; Hughes, L.; Dong, Y.; Barwich, S.; Vaesen, S.; Shvets, I. V.; Möbius, M.; Schmitt, W.; Gogotsi, Y.Nicolosi, V. 4D printing of MXene hydrogels for high-efficiency pseudocapacitive energy storage. *Nat. Commun.* **2022**, 13, 6884.
17. Fan, Z.; Yang, Y.; Ma, H.; Wang, Y.; Xie, Z.Liu, Y. High-volumetric capacitance and high-rate performance in liquid-mediated densified holey MXene film. *Carbon*. **2022**, 186, 150-159.
18. Dutta, P.; Patra, A.; Deb, S. K.; Sikdar, A.; Majumdar, A.; Karim, G. M.Maiti, U. N. Freestanding MXene-hydrogels prepared via critical density-controlled self-assembly: high-performance energy storage with ultrahigh capacitive vs. diffusion-limited contribution. *J. Mater. Chem. A*. **2021**, 9, 25013-25023.
19. Bayram, V.; Ghidiu, M.; Byun, J. J.; Rawson, S. D.; Yang, P.; McDonald, S. A.; Lindley, M.; Fairclough, S.; Haigh, S. J.; Withers, P. J.; Barsoum, M. W.; Kinloch, I. A.Barg, S. MXene Tunable Lamellae Architectures for Supercapacitor Electrodes. *ACS Appl. Energy Mater.* **2020**, 3, 411-422.
20. Halim, J.; Kota, S.; Lukatskaya, M. R.; Naguib, M.; Zhao, M.-Q.; Moon, E. J.; Pitock, J.; Nanda, J.; May, S. J.; Gogotsi, Y.Barsoum, M. W. Synthesis and Characterization of 2D Molybdenum Carbide (MXene). *Adv. Funct. Mater.* **2016**, 26, 3118-3127.
21. Tang, J.; Mathis, T.; Zhong, X.; Xiao, X.; Wang, H.; Anayee, M.; Pan, F.; Xu, B.Gogotsi, Y. Optimizing Ion Pathway in Titanium Carbide MXene for Practical High-Rate Supercapacitor. *Adv. Energy Mater.* **2021**, 11, 2003025.
22. Li, L.; Zhang, M.; Zhang, X.Zhang, Z. New Ti3C2 aerogel as promising negative electrode materials for asymmetric supercapacitors. *J. Power Sources*. **2017**, 364, 234-241.

23. Dutta, P.; Sikdar, A.; Majumdar, A.; Borah, M.; Padma, N.; Ghosh, S.Maiti, U. N. Graphene aided gelation of MXene with oxidation protected surface for supercapacitor electrodes with excellent gravimetric performance. *Carbon*. **2020**, 169, 225-234.

24. Yao, M.; Chen, Y.; Wang, Z.; Shao, C.; Dong, J.; Zhang, Q.; Zhang, L.Zhao, X. Boosting gravimetric and volumetric energy density via engineering macroporous MXene films for supercapacitors. *Chem. Eng. J.* **2020**, 395, 124057.

25. Fan, Z.; Wang, Y.; Xie, Z.; Xu, X.; Yuan, Y.; Cheng, Z.Liu, Y. A nanoporous MXene film enables flexible supercapacitors with high energy storage. *Nanoscale*. **2018**, 10, 9642-9652.

26. Cai, Z.; Ma, Y.-F.; Wang, M.; Qian, A. N.; Tong, Z.-M.; Xiao, L.-T.; Jia, S.-T.Chen, X.-Y. Engineering of electrolyte ion channels in MXene/holey graphene electrodes for superior supercapacitive performances. *Rare Metals*. **2022**, 41, 2084-2093.

27. Zhang, P.; Zhu, Q.; Soomro, R. A.; He, S.; Sun, N.; Qiao, N.; Xu, B. In Situ Ice Template Approach to Fabricate 3D Flexible MXene Film-Based Electrode for High Performance Supercapacitors. *Adv. Funct. Mater.* **2020**, 30, 2000922.

28. Wang, G.; Jiang, N.; Xu, Y.; Zhang, Z.; Wang, G.; Cheng, K. Solvent-assisted assembly of reduced graphene oxide/MXene-polypyrrole composite film for flexible supercapacitors. *J. Colloid Interface Sci.* **2023**, 630, 817-827.

29. Zhang, L.; Or, S. W. Self-assembled three-dimensional macroscopic graphene/MXene-based hydrogel as electrode for supercapacitor. *APL Materials*. **2020**, 8, 091101.

30. Ran, F.; Wang, T.; Chen, S.; Liu, Y.; Shao, L. Constructing expanded ion transport channels in flexible MXene film for pseudocapacitive energy storage. *Appl. Surf. Sci.* **2020**, 511, 145627.

31. Enaiet Allah, A. Three-dimensional N-doped mesoporous carbon–MXene hybrid architecture for supercapacitor applications. *RSC Adv*. **2023**, 13, 9983-9997.
